# Systems Perspective of Amazon Mechanical Turk for Organizational Research: Review and Recommendations

**DOI:** 10.3389/fpsyg.2017.01359

**Published:** 2017-08-08

**Authors:** Melissa G. Keith, Louis Tay, Peter D. Harms

**Affiliations:** ^1^Department of Psychological Sciences, Purdue University West Lafayette, IN, United States; ^2^Department of Management, The University of Alabama Tuscaloosa, AL, United States

**Keywords:** mechanical turk, sampling, review, meta-analysis, research design

## Abstract

Amazon Mechanical Turk (MTurk) is becoming a prevalent source of quick and cost effective data for organizational research, but there are questions about the appropriateness of the platform for organizational research. To answer these questions, we conducted an integrative review based on 75 papers evaluating the MTurk platform and 250 MTurk samples used in organizational research. This integrative review provides four contributions: (1) we analyze the trends associated with the use of MTurk samples in organizational research; (2) we develop a systems perspective (recruitment system, selection system, and work management system) to synthesize and organize the key factors influencing data collected on MTurk that may affect generalizability and data quality; (3) within each factor, we also use available MTurk samples from the organizational literature to analyze key issues (e.g., sample characteristics, use of attention checks, payment); and (4) based on our review, we provide specific recommendations and a checklist for data reporting in order to improve data transparency and enable further research on this issue.

The recent past has seen a steady increase in the number of published studies utilizing Amazon's Mechanical Turk (MTurk) platform for crowdsourcing participants and workers (Harms and DeSimone, 2015; Bohannon, 2016). Researchers and businesses can pay a fee to the MTurk online community (i.e., “workers” or “turkers”) to complete Human Intelligence Tasks (HITs). HITs may include surveys, experiments, coding tasks, or any other work requiring human intelligence. The ease of data collection and relatively lower cost of collecting data through MTurk as compared to traditional survey methods, and even online survey companies, has created a unique opportunity for the field of organizational science to collect research data at a substantially reduced cost.

The opportunity afforded by MTurk has led to an increasing use of the platform by organizational researchers. In 2015, 63 studies using MTurk samples were published in 11 organizational journals (i.e., *Academy of Management Journal, Administrative Science Quarterly, Journal of Applied Psychology, Journal of Business and Psychology, Journal of Organizational Behavior, Journal of Vocational Behavior, Leadership Quarterly, Organizational Behavior and Human Decision Processes, Organizational Research Methods, Organization Science*, and *Personnel Psychology*) compared to just seven published studies in the same journals in 2012. This trend suggests that within the next few years we will see hundreds of studies within organizational journals that utilize MTurk samples. Despite the growing use of MTurk samples within organizational and psychological research, some scholars have raised concerns about its use and current debates surround whether MTurk is appropriate for organizational research (see Berinsky et al., 2012; Goodman et al., 2012; Crump et al., 2013; Harms and DeSimone, 2015; Cheung et al., 2016; Keith and Harms, 2016).

The issue of whether MTurk samples are appropriate for the study of organizational behavior is a critical one given its growing use in organizational science (and related fields). Specifically, there is a need to address whether the various concerns often raised by editors, reviewers, and researchers should outweigh the convenience and reduced cost (Rosenthal, 1994). Despite the rhetoric surrounding MTurk, to our knowledge, a systematic review examining the literature on MTurk within organizational research that addresses these questions and provides specific recommendations is absent. To shed light on MTurk as a sampling platform, we present an integrative overview of the potential issues surrounding the use of MTurk by conducting a review of past qualitative and quantitative research evaluating MTurk samples and methodology, as well as, a review of organizational research utilizing MTurk samples.

We make four key contributions to research, methods, and practice. First, we provide a detailed overview of the MTurk platform to provide researchers with an understanding of the nature of the MTurk platform and resources related to MTurk data collection. We analyze key trends associated with MTurk usage in organizational research based on the past decade of studies conducted using MTurk samples. Second, we develop a systems framework (recruitment system, selection system, and work management system) to synthesize and organize past research. Using this framework, researchers can understand how specific research choices made within these systems influence the generalizability and quality of data collected on MTurk. We propose that the broader systems perspective taken here can be applied to understand key issues in other types of samples and data collection (e.g., student samples, online panels, community samples, field samples). Third, within this framework, we use available MTurk studies from the organizational literature to analyze key issues (e.g., reporting practices, current uses). Therefore, this review empirically establishes answers to important questions. Fourth, based on our review, we present best practices and recommendations for researchers and include a detailed checklist for MTurk data collection reporting to increase data transparency.

## Overview of MTurk

Amazon launched MTurk in 2005 for internal work that required human intelligence. Since then, MTurk has grown immensely and is open to anyone who registers for an Amazon account. Individuals may utilize MTurk as a “worker” (an individual who participates in human intelligence tasks or “HITs”) or a “requestor” (an individual who posts the HITs). Each worker is identified with a unique worker ID, which remains constant unless the worker creates a new account.

A requestor can post a HIT using the internal MTurk survey platform or by posting a link to an external survey platform (e.g., Qualtrics, Survey Monkey). A HIT may be a survey, a coding task, or any other task that requires human intelligence. Requestors must also decide upon a title, description, and keywords to describe their HIT, the payment per HIT, the number of workers that may complete a HIT, the time allotted for each HIT, the expiration date for the HIT, and the worker requirements (qualifications). The HIT can be released in batches, which allows for an easy test run before collecting a large amount of data. That is, a requestor can release a small batch of HITs to obtain feedback on the HIT or be sure the data collection is running smoothly.

When searching for HITs, Amazon defaults to show the newest HITs; however, workers may also sort the HITs by the oldest HITs, fewest (or most) HITs available, payment, expiration data, title (A–Z or Z–A), and time allotted. Workers are able to search for particular keywords or qualifications and specify a minimum amount of payment. Past research has suggested that workers tend to search for HITs based on which are the most recent (the default) and by the number of HITs available within a task (Chilton et al., 2010).

After the HITs are completed, the requestor must either accept or reject the work. This can either be done manually or automatically by setting an “auto approval delay.” Once the work is accepted, the worker is compensated through their Amazon account.

More recently, third party platforms (e.g., psiTurk, TurkGate, TurkPrime) have emerged to make MTurk more “researcher friendly.” The most comprehensive platform as of 2017 is TurkPrime (see Litman et al., 2016 for an excellent summary of the platform; http://www.turkprime.com/), which permits greater researcher control and flexibility without requiring extensive programming knowledge. To use TurkPrime, a requestor simply links their created TurkPrime account to an existing MTurk account. We view TurkPrime as a valuable resource for researchers and integrate its uses into our review where appropriate.

## Literature search

To examine the issues related to the MTurk platform and use of MTurk samples, we conducted a literature search for qualitative and quantitative research evaluating MTurk samples and methodology. Our search was broad in scope and covered past research reviewing the use of MTurk within organizational research and among allied fields such as psychology, education, and sociology. Therefore, we conducted a literature search in the Business Source Premier, Education Full Text, ERIC, PsycARTICLES, PsycINFO, and Social Sciences databases using the following keywords: “MTurk” OR “Mechanical Turk.” This initial search yielded a total of 371 articles. Articles that were included in our review involved one or more of the following topics: (1) offering reviews or tutorials of the MTurk platform, (2) examining the quality of data collected on MTurk, or (3) examining various characteristics of MTurk samples, particularly in relation to other samples. Only articles focusing on MTurk for use by researchers were considered in the current review; that is, articles not directly examining the MTurk platform and participants were excluded from the current review. Based on these criteria, we found 75 relevant articles.

To supplement our initial search and to obtain information about the usage of MTurk samples within organizational research, we also conducted a manual search of every article published between 2005 and 2015 in the following journals: *Academy of Management Journal (AMJ), Administrative Science Quarterly (ASQ), Journal of Applied Psychology (JAP), Journal of Business and Psychology* (*JBP*), *Journal of Management (JOM), Journal of Occupational and Organizational Psychology* (*JOOP*), *Journal of Organizational Behavior (JOB), Journal of Vocational Behavior (JVB), Leadership Quarterly* (*LQ*), *Organizational Behavior and Human Decision Processes (OBHDP), Organizational Research Methods (ORM), Organization Science* (*OS*), and *Personnel Psychology (PPsych*). These journals represent top journals in the field of organization science (Zickar and Highhouse, 2001; Podsakoff et al., 2005), and give a good indication of how MTurk is currently being utilized in the organizational sciences. We also requested unpublished studies on the human resources (HRDIV_NET) and organizational behavior (OB-LIST) listservs. Notably, our review did not find MTurk samples prior to 2012. The only inclusion criterion for the current review was that the article had to possess at least one sample from the MTurk subject pool. The search yielded 138 articles utilizing 250 MTurk samples in all the journals sampled with the exception of *Journal of Occupational and Organizational Psychology* (see Table 1).

**Table 1:** Summary of organizational journals and unpublished manuscripts with MTurk samples.

**Journal**	**No. of**	**No. of**	**No. of**
	**Articles**	**Samples total**	**MTurk samples**
*Academy of Management Journal*	4	12	5
*Administrative Science Quarterly*	1	4	1
*Journal of Applied Psychology*	15	53	21
*Journal of Business and Psychology*	10	23	10
*Journal of Management*	6	21	9
*Journal of Organizational Behavior*	5	14	6
*Journal of Occupational and Organizational Psychology*	0	–	–
*Journal of Vocational Behavior*	1	2	1
*Leadership Quarterly*	12	43	17
*Organizational Behavioral and Human Decision Processes*	66	295	156
*Organizational Research Methods*	4	10	6
*Organization Science*	5	12	5
*Personnel Psychology*	3	11	5
Other^*^	6	11	8
Total	138	511	250

* *Other includes unpublished manuscripts, conference papers, or submitted papers not in one of the sampled journals*.

## MTurk trends in the organizational sciences

Our literature search revealed wide and increasing interest in MTurk. Despite the relatively recent introduction, it has garnered a great deal of attention and scrutiny in a variety of fields apart from the organizational sciences including, but not limited to, behavioral economics (Horton et al., 2011; Wolfson and Bartkus, 2013), social psychology (Summerville and Chartier, 2013), cognitive psychology (Crump et al., 2013), clinical psychology (Shapiro et al., 2013; Arditte et al., 2016), and political science (Berinsky et al., 2012).

Based on our search of published work utilizing MTurk samples in the organizational sciences, we can make several broad statements concerning how organizational researchers currently use MTurk. First, we note that most (93.5%) of the articles included in these analyses were multiple study papers (*M* = 3.70, *SD* = 1.74, Mode = 4). Approximately half of these studies were MTurk samples (*M* = 1.80, *SD* = 1.21). That is, on average, articles containing at least one MTurk sample had around four samples in total and approximately two MTurk samples. Only four articles in the surveyed articles were single study papers (Karim and Behrend, 2014; Karim et al., 2014; Credé and Harms, 2015; Fine and Pirak, 2016); however, our request for unpublished studies found other single study papers. Based on these findings and the relative ease of collecting data quickly on MTurk, we speculate that the increasing pressure for replication (Pashler and Harris, 2012; Klein et al., 2014; Stanley and Spence, 2014; Maxwell et al., 2015) may be a partial cause of MTurk's increasing popularity. We add that although some research has been published in top management journals using only MTurk samples (i.e., Barber et al., 2013; DeKay et al., 2014; Karim and Behrend, 2014; Karim et al., 2014; Credé and Harms, 2015; Proudfoot et al., 2015; Saqib and Chan, 2015; Savani and King, 2015; Fine and Pirak, 2016), this is relatively rare as only 9 out of 138 published articles exclusively used MTurk samples.

**Figure 1:**
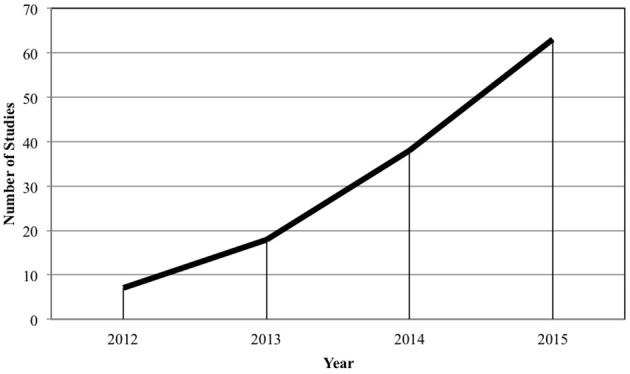
Studies publishing MTurk samples in the organizational sciences 2012–2015.

Second, as shown in Figure 1, we observed a steady, upward trend in published research incorporating an MTurk sample. Published studies utilizing MTurk samples have increased by 800% between 2012 and 2015. Given this steady increase, it is essential for organizational researchers to be informed about both the opportunities and challenges presented by this platform.

Third, out of the 138 articles reviewed, 66 (47.83%) were published in *OBHDP*. Additionally, *OBHDP* accounts for 156 of the 250 MTurk samples examined in our quantitative review, as seen in Table 1. We speculate that *OBHDP* articles often contain papers utilizing both experiments and multiple studies, and MTurk samples are a convenient means of running experiments and conducting replications. To illustrate, our sample of *OBHDP* articles had an average of 4.47 (*SD* = 1.74) studies per article. Furthermore, ~40% of the articles we reviewed for the purposes of this paper from *OBHDP* contained five or more studies in the article (16.7% were seven or eight study articles). This finding can be compared to *JAP*, which had the second highest frequency of MTurk samples. Approximately 20% of the *JAP* articles containing at least one MTurk sample had five or six studies total; however, none had more than six studies. This suggests that MTurk may be considered a viable option for organizational researchers who wish to replicate findings with a different sample quickly.

## A systems perspective for evaluating mechanical turk

Based on the literature, we organized the research into three broad systems that examine MTurk through an organizational science lens. We argue that MTurk (as well as other samples used in the social sciences) can be viewed as a labor market that has an extant labor force from which to recruit, select, and manage work. Specifically, we examine MTurk as a recruitment system, a selection system, and a work management system. Each of these systems is made up of a unique set of factors that influence generalizability and data quality. We examine generalizability and data quality in particular because these criteria are highly relevant to our ability to draw inferences as researchers. For MTurk to be a viable option for organizational researchers, the sample should be representative of the target population (e.g., employed workers; Bergman and Jean, 2016) and appropriate for the research questions within organizational settings. Additionally, we must be able to trust that the data provided by MTurk workers is accurate, complete, and psychometrically sound (for psychometrically validated scales). Summary tables for each system and its factors can be found in the Supplementary Material (Supplementary Tables 1–3).

### MTurk as a recruitment system

Accumulated organizational research shows that the extant pool of job candidates determines the effectiveness of selection procedures and the eventual workforce (Breaugh, 2008; Newman and Lyon, 2009). Likewise, MTurk comprises an extant pool of workers who have preexisting characteristics that are recruited to complete surveys. As will be discussed later, the researcher should discern whether MTurk participants are appropriate for the research question and research design (Highhouse and Gillespie, 2009). To add complexity, MTurk workers have a great deal of autonomy to enter, leave, or return to the workforce, and may participate in as many HITs as they are qualified for.

#### Worker characteristics

A common question posed by researchers is, “Who are the MTurk workers?” As such, a great deal of empirical work has examined sample characteristics. In particular, MTurk sample characteristics have been compared to student samples (Paolacci et al., 2010; Behrend et al., 2011; Berinsky et al., 2012; Goodman et al., 2012; Johnson and Borden, 2012; Casler et al., 2013; Steelman et al., 2014; Feitosa et al., 2015; Roulin, 2015), community samples (Berinsky et al., 2012; Goodman et al., 2012; Feitosa et al., 2015), Internet panels (Paolacci et al., 2010; Berinsky et al., 2012; Steelman et al., 2014; Roulin, 2015), social media (Casler et al., 2013), and nationally representative samples (Berinsky et al., 2012; Simons and Chabris, 2012). We integrate these findings to provide a summary of these results in Table 2. Further, we provide a summary of sample characteristics from the MTurk samples drawn from the organizational research in Table 3.

**Table 2:** Summary of comparisons between MTurk samples and other samples.

**Characteristic**	**MTurk**	**Student**	**Community/field**	**Other internet**	**Social media**	**Nationally representative surveys**
*K*	13	9	5	4	1	4
*N* (range)	32–998	24–1,428	60–263	137–3,003	30	1838–308,745,538
*M* Age	31.9^1^	20.92^1^	41.55^16^	36.88^20^	26^24^	46.8^9^
% 18–24	30.52^2^	–	–	–	–	7.93^2^
% 25–34	30.11^2^	–	–	–	–	11.35^2^
% 35–44	18.51^2^	–	–	–	–	12.95^2^
% 45–54	12.41^2^	–	–	–	–	18.22^2^
% 55–64	7.02^2^	–	–	–	–	18.83^2^
% 65–74	0.92^2^	–	–	–	–	14.83^2^
% over 75	0.51^2^	–	–	–	–	15.91^2^
**GENDER**						
Male	49.25^3^	40.63^11^	32.78^17^	45.95^21^	17^24^	44.8^25^
Female	50.68^3^	56.60^11^	67.22^17^	53.55^21^	83^24^	55.20^25^
**RACE/ETHNICITY**						
% White	55.82^4^	67.98^12^	81.43^18^	82^22^	93^24^	78.08^25^
% Black	5.48^4^	9.69^13^	12.77^18^	7.45^22^	–	10.73^25^
% Hispanic	4.05^5^	5.71^14^	3^19^	5.5^22^	3.5^24^	10.05^25^
% Asian	35.32^6^	9.26^14^	1.5^19^	7^15^	3.5^24^	1.67^2^
% Other	5.94^6^	3.53^14^	2.3^19^	3^15^	–	6.87^2^
**EDUCATION**						
% No college	16.99^7^	–	24.3^19^	16^15^	–	28.04^2^
% Some college	25.61^7^	–	25.9^19^	39^15^	–	37.31^2^
% College degree	38.11^8^	–	37.6^19^	37.5^23^	–	21.34^2^
% Advanced degree	22.46^7^	–	9.9^19^	18^15^	–	13.31^2^
*M* Income	55,332^9^	–	–	69,043^9^	–	62,378.50^9^
% <$40,000	67.94^10^	86^15^	–	29^15^	–	51.07^2^
% $40–80,000	22.17^10^	5^15^	–	37^15^	–	32.35^2^
% >$80,000	9.64^10^	5^15^	–	32^15^	–	16.58^2^

Demographic averages taken from the following samples:

1 *Paolacci et al., 2010; Berinsky et al., 2012; Goodman et al., 2012; Johnson and Borden, 2012; Casler et al., 2013; Steelman et al., 2014; Roulin, 2015*.

2 *Simons and Chabris, 2012*.

3 *Paolacci et al., 2010; Behrend et al., 2011; Berinsky et al., 2012; Goodman et al., 2012; Johnson and Borden, 2012; Simons and Chabris, 2012; Casler et al., 2013; Steelman et al., 2014; Feitosa et al., 2015; Roulin, 2015*.

4 *Behrend et al., 2011; Berinsky et al., 2012; Johnson and Borden, 2012; Simons and Chabris, 2012; Casler et al., 2013; Steelman et al., 2014; Feitosa et al., 2015; Roulin, 2015*.

5 *Behrend et al., 2011; Berinsky et al., 2012; Simons and Chabris, 2012; Casler et al., 2013; Steelman et al., 2014; Feitosa et al., 2015; Roulin, 2015*.

6 *Behrend et al., 2011; Simons and Chabris, 2012; Casler et al., 2013; Steelman et al., 2014; Feitosa et al., 2015; Roulin, 2015*.

7 *Behrend et al., 2011; Simons and Chabris, 2012; Steelman et al., 2014; Feitosa et al., 2015*.

8 *Behrend et al., 2011; Simons and Chabris, 2012; Steelman et al., 2014; Feitosa et al., 2015; Roulin, 2015*.

9 *Berinsky et al., 2012*.

10 *Simons and Chabris, 2012; Steelman et al., 2014*.

11 *Behrend et al., 2011; Berinsky et al., 2012; Casler et al., 2013; Feitosa et al., 2015: Goodman et al., 2012; Johnson and Borden, 2012; Steelman et al., 2014; Roulin, 2015*.

12 *Goodman et al., 2012; Johnson and Borden, 2012; Steelman et al., 2014; Roulin, 2015*.

13 *Johnson and Borden, 2012; Casler et al., 2013; Steelman et al., 2014; Feitosa et al., 2015; Roulin, 2015*.

14 *Casler et al., 2013; Feitosa et al., 2015; Roulin, 2015*.

15 *Steelman et al., 2014*.

16 *Berinsky et al., 2012; Goodman et al., 2012*.

17 *Berinsky et al., 2012; Goodman et al., 2012; Feitosa et al., 2015*.

18 *Berinsky et al., 2012; Feitosa et al., 2015*.

19 *Feitosa et al., 2015*.

20 *Paolacci et al., 2010; Berinsky et al., 2012; Roulin, 2015*.

21 *Paolacci et al., 2010; Berinsky et al., 2012; Steelman et al., 2014; Roulin, 2015*.

22 *Berinsky et al., 2012; Steelman et al., 2014*.

23 *Steelman et al., 2014; Roulin, 2015*.

24 *Casler et al., 2013*.

25 *Berinsky et al., 2012; Simons and Chabris, 2012*.

**Table 3:** Reports of MTurk sample characteristics in the organizational literature.

	**No. of Samples reporting**	**% Samples reporting**	***M* (*SD*)**	**Range**
Sample size	248	99.20	240.51 (206.05)	18–1309
Mean age	198	79.20	33.06 (3.03)	20–47.6
% Male^a^	208	83.53	50.82 (9.56)	26–72.4
% College education^b^	37	14.86	64.65 (12.96)	39–92
% White^c^	67	27.92	74.48 (11.71)	15.3–90
% Full time employed^d^	11	5.29	50.63 (14.83)	28.3–72
% Part time employed^d^	6	2.88	25.40 (4.47)	18.5–30
% Unemployed^d^	8	3.85	25.75 (7.33)	15.9–38

*Total number of samples is 250*.

a Filtered out samples selecting for gender (n = 1);

b Filtered out samples selecting for college education (n = 1);

c Filtered out studies selecting for race (n = 10);

d *Filtered out studies selecting for employment (n = 42)*.

*% Samples reporting reflects total number of samples minus any filtered out studies*.

##### Generalizability

Our summary of results in Table 2 show that MTurk samples tend to be more diverse in terms of education and age than college samples. Compared to community samples, MTurk samples appear to be younger and more educated; however, past research has not found these differences to be statistically significant (Goodman et al., 2012). Not surprisingly, MTurk is the most similar to other Internet samples with the exception of racial distribution (Berinsky et al., 2012). If not limited to the U.S., MTurk workers are likely to be ethnically diverse (Buhrmester et al., 2011); however, the sample tends to be anchored primarily in the U.S. or India (Ipeirotis, 2010). Overall, MTurk samples are more diverse than regular college samples and more similar to other Internet samples.

When comparing MTurk samples (restricted to the U.S.) to nationally representative samples (e.g., U.S. Census, Survey USA), MTurk samples are younger, more educated, and lower on the income scale. This demonstrates that MTurk samples are not representative of the United States. However, this is not a surprising finding given that college, Internet, and community samples are not expected to be representative of the United States.

Table 3 shows demographics of MTurk samples used in organizational research. These results are consistent with results from Table 2. We find that samples are usually older than college-aged samples. Further, half of the MTurkers are employed part-time or unemployed. This is consistent with past findings (e.g., Ross et al., 2010; Behrend et al., 2011). A population of unemployed or underemployed participants offers a potentially interesting sampling opportunity for research questions related to job search behavior, underemployment, and unemployment (Roulin, 2015). At the same time, this could explain why MTurkers have a lower income on average as compared to nationally representative samples.

Taken together, our conclusions echo the conclusions of other researchers (cf., Behrend et al., 2011; Buhrmester et al., 2011; Berinsky et al., 2012; Casler et al., 2013; Brandon et al., 2014; Woo et al., 2015): MTurk samples are *not* representations of the general population. We should carefully consider whether representativeness of national data should be the criterion, as organizational researchers have traditionally collected data from class settings (e.g., MBA or undergraduate), specific organizations/communities, and even other online panel data from survey companies where participant solicitation processes are not transparent. Within organizational research, it may be more important to ask whether MTurk demographic characteristics are appropriate for the specific study goals at hand (Landers and Behrend, 2015). The issue of selection within the broader MTurk worker pool is also critical given that researchers may only be interested in MTurkers who are employed full-time.

Also relevant to organizational researchers is past research examining the self-reported professions of the workers (e.g., Downs et al., 2010; Behrend et al., 2011; Harms and DeSimone, 2015). The conclusion to be drawn from these studies is that MTurk participants come from a diverse set of industries, ranging from STEM (Science, Technology, Engineering, and Mathematics) to the arts. We can compare this to Shen et al.'s (2011) examination of *JAP* samples, which found that 71.13% of the samples published utilized homogenous samples in terms of job type. Thus, MTurk offers opportunity for examining a wider range of occupations, particularly occupations previously understudied (Smith et al., 2015; Bergman and Jean, 2016).

With regard to measures of individual differences, researchers have compared MTurk samples to other samples on Big 5 personality dimensions and clinical symptoms. Goodman et al. (2012) compared MTurk participants to a community sample and a student sample resulting in a common theme that MTurk participants were significantly lower on extraversion, emotional stability, and self-esteem. Similarly, Behrend et al. (2011) found that MTurk workers were significantly lower on extraversion compared to the student sample. Researchers have also found higher levels of anxiety and depression in MTurk samples (Shapiro et al., 2013; Arditte et al., 2016). For example, Shapiro et al. (2013) reported that social anxiety in MTurk samples is seven times higher than in the general population. Attitudes and emotions of MTurk workers have also been compared to other samples revealing that MTurk workers report lower life satisfaction compared to other samples (Shapiro et al., 2013). Grysman (2015) also found that MTurk participants are more likely to perceive stressful events as more difficult than college students. Thus, there appears to be a trend where MTurk participants are less emotionally stable, have higher negative affect, and lower levels of well-being than the other samples or the general population. This suggests that MTurk workers have a large degree of negative attitudes/emotions.

##### Data quality

One of the primary concerns surrounding MTurk has been whether it is possible to obtain quality data. In other words, is the data collected on MTurk complete, accurate, and do MTurk samples produce psychometric scale properties similar to other samples? With regards to completeness, some research suggests that MTurk samples are less likely to complete a survey compared to student samples (Bartneck et al., 2015; Zhou and Fishbach, 2016). More recently, Zhou and Fishbach (2016) collected 88 MTurk samples (*N* = 22,260 individual participants) and found that more than 20% of the 88 studies had a dropout rate greater than 30%. In contrast, the group also collected 82 student (lab) samples (*N* = 7,861) finding that 96% had no attrition. However, the key issue may be less so the differences in sample but the mode of data collection. Behrend et al. (2011) found no significant differences between student participants and MTurk participants when studies were administered online. Dropout rates of similar amounts are generally expected in online panel surveys, and many online panel recruitment companies account for this by providing a larger initial sample to meet the targeted sample size.

Accuracy has been raised as a potential concern with MTurk samples. Past research suggests that MTurk samples may be prone to inattention (Chandler et al., 2014), social desirability bias (Behrend et al., 2011; Antin and Shaw, 2012), and dishonesty (Rand, 2012; Shapiro et al., 2013; Peer et al., 2014; Daly and Nataraajan, 2015). Because there is no way to control the environment that individuals are in while completing HITs, there is the possibility that workers are distracted by surrounding stimuli (e.g., television) or may be completing multiple HITs at once (Chandler et al., 2014). Additionally, MTurk workers may seek to please requestors who are paying for their work and respond in a way that is more socially desirable than student samples (Behrend et al., 2011). Past research has also revealed that MTurk workers are not always honest. Peer et al. (2014) found that 36% of low-reputation workers (those possessing an approval rating under 95%) self-reported a reputation at or above 95%. Further, dishonesty and inaccuracies have been found in workers reporting seemingly innocuous information such as gender, year of birth, location, education level, or income level (Rand, 2012; Shapiro et al., 2013; Daly and Nataraajan, 2015). While this seems to suggest that data collected from MTurk are problematic, there are a couple of significant caveats. First, with regard to honesty, the number of workers reporting different genders, ages, etc. between waves of data collection were a small minority in these studies, typically no higher than 6% for items such as age, gender, and location, and no higher than 19% for items such as education level and income. There is also a possibility that more than one individual uses the same Amazon account to complete HITs on MTurk. Second, these MTurk studies do not make a comparison to other samples such as other online panel data, student samples, or organizational data, in which the same problem of potential dishonesty/inaccuracies are also prevalent (Meade and Craig, 2012). Third, other research suggests that MTurk participants are just as accurate in their ability to recall previously seen items and are more attentive to instructions than undergraduate participants (Hauser and Schwarz, 2015; Ramsey et al., 2016). Overall, there is no clear evidence that MTurk samples are always less accurate compared to other samples. More research is needed comparing MTurk samples to other samples.

With regard to responses on psychological scales, MTurk data have been shown to be psychometrically sound, suggesting that data are of good quality. Reliability has been demonstrated in MTurk samples in the form of acceptable internal consistency (Behrend et al., 2011; Johnson and Borden, 2012; Bates and Lanza, 2013; Shapiro et al., 2013; Steelman et al., 2014; Rouse, 2015; Schleider and Weisz, 2015), test-retest reliability (Buhrmester et al., 2011; Holden et al., 2013; Daly and Nataraajan, 2015; Schleider and Weisz, 2015), interrater reliability within the sample (Conley and Tosti-Kharas, 2014; Costa-jussà et al., 2014), interrater agreement within the sample (Bartneck et al., 2015), and interrater reliability with experts (Alonso and Mizzaro, 2012; Conley and Tosti-Kharas, 2014; Costa-jussà et al., 2014). MTurk has also demonstrated measurement equivalence with other samples including student and employee samples (Behrend et al., 2011; Steelman et al., 2014; Feitosa et al., 2015). Not surprisingly, MTurk samples from non-native English speaking countries do not fair as well psychometrically, particularly in terms of measurement equivalence (Feitosa et al., 2015).

##### Recommendations

While researchers can select and prescreen research participants from MTurk, it is important for researchers to understand the characteristics of the general MTurk participant pool. It is more diverse compared to student samples, and a substantial number of MTurkers are from other countries (e.g., India). Within the United States, they are not representative of the general U.S. population and a large proportion of MTurkers reported being unemployed, are likely underemployed, and have higher negative emotions and attitudes. Therefore, when conducting a study using MTurk, it is important for organizational researchers to determine if the general MTurk population is appropriate. It is likely that selection and screening procedures will need to be implemented to obtain the appropriate participants (see subsequent sections). In general, there does not appear to be strong evidence that MTurk participants produce lower quality data. However, researchers should be aware of the dropout rates (20–30%) and need to account for this in their budgeting and research designs (online survey participation in general). In addition, researchers need to be aware that the large portion of non-English speakers in the MTurk pool that may potentially affect results (e.g., reliability and validity).

#### Super turkers

According to Amazon, there are more than 500,000 registered workers from 190 countries (https://requester.mturk.com/tour). However, registered workers may not equate to active workers, and active workers may not equate to survey takers. There is some concern that “professional workers” or “Super Turkers” complete the majority of the organizational/psychological studies on MTurk (Fort et al., 2011; Stewart et al., 2015).

There is evidence for the presence of Super Turkers based on estimates of active survey takers and repeat participation. Past research using open-population, capture-recapture analysis has estimated that the actual survey-taking population on MTurk is estimated to be around 7,300 workers. Similarly Chandler et al. (2014) combined MTurk samples from authors and collaborators and found that out that 7,498 workers were responsible for completing 16,408 HITs from 132 batches. Indicative of Super Turkers, repeat participation in similar research also seems to occur regularly on MTurk. Over the course of 3 months, 36% of the workers sampled completed survey HITs from more than one laboratory (Stewart et al., 2015). Similarly, Fort et al. (2011) estimated that 80% of the survey HITs are being completed by 20% of the most active workers. Further, the most active 1 and 10% of workers are estimated to be responsible for completing 11 and 41% of the HITs, respectively.

##### Generalizability

The presence of Super Turkers suggests that active survey workers may view MTurk as a full-time job or may view their full-time work in significantly different ways from workers who do not actively engage in paid survey responding. For example, Brawley and Pury (2016) found that U.S. workers who viewed MTurk as their primary job, on average, work nearly twice as many hours per week on MTurk (*M* = 35.22, *SD* = 16.65), compared to U.S. workers who do not view MTurk as their primary job (*M* = 18.25, *SD* = 11.12). These results demonstrate that MTurk workers can view MTurk as a job, and the workers viewing MTurk as a primary job are more likely to be represented in surveys.

##### Data quality

Super Turkers may not pose a problem to survey completeness or psychometric soundness *per se*. However, repeated participation in similar experiments may pose a problem for the accuracy of conclusions drawn from MTurk samples due to exposure effects. To illustrate, 59 and 52% of workers self-reported exposure to the prisoner's dilemma and ultimatum paradigms, respectively (Chandler et al., 2014). There are three ways exposure could affect data quality (cf. Stewart et al., 2015): (1) workers may be less responsive to common experimental manipulations due to learning or practice effects; (2) exposure to debriefing materials may lead workers to infer demand characteristics on similar experiments; and (3) workers may inflate measures of ability due to practice effects.

##### Recommendations

It is important for researchers to recognize that the regular survey-taking population on MTurk is likely closer to 7,300 workers rather than 500,000 workers, which would affect the extent to which large scale surveys can be conducted. Further, the pool of workers may be even smaller within the United States. Researchers should make efforts to reduce repeat participation across studies by assigning a qualification to each worker ID (Peer et al., 2012), monitoring worker IDs across studies, or by using TurkPrime (see Litman et al., 2016). Given non-naïveté can reduce effect sizes in experimental designs (Chandler et al., 2015), we suggest considering the nature of the research design and weighing the risk of non-naïveté.

With the presence of Super Turkers, organizational researchers seeking to survey an employed sample on MTurk should include items asking about the nature of the work rather than a simple demographic item asking whether the worker is employed. Super Turkers may consider MTurk to be a full-time job, which may not be appropriate for addressing certain organizational questions.

### MTurk as a selection system

MTurk is also a selection system whereby researchers can use criteria to select workers, namely, qualifications and prescreens. Requestors can screen out workers who do not meet the qualifications for the work or fail to perform well on a selection test. Similar to selection mechanisms and systems in organizations (Schneider, 1987), MTurk workers also self-select into HITs based upon initial attraction to that HIT. On MTurk, attraction will be driven by factors such as compensation and the perceived nature of the task. Once the worker self-selects into the HIT, the worker may then self-select out of a HIT. Below we detail how qualifications, prescreens, and self-selection differentially impact generalizability and data quality.

#### Qualifications

Setting qualifications will limit the availability of the HIT to qualified MTurk workers. MTurk allows researchers to specify up to five qualifications. Standard qualifications that researchers can choose from include HIT approval rate, number of HITs approved, and location. The HIT approval rate specifies the percentage of completed work that has been approved (as opposed to denied) by other requestors. Researchers can specify the range of approval percentage desired ranging from 0 to 100. Researchers can also specify the number of HITs approved, which is essentially a qualification that stipulates the level of experience on MTurk. Finally, researchers can specify the location of the workers to be of a particular country or even a specific state(s) within the United States. In addition to the standard qualifications, custom qualifications can also be made.

How are qualifications used and reported within organizational research? We found a lack of homogeneity in whether qualifications were used and the number of qualifications used. Out of 250 samples used in organizational research, about half of the samples (52%) reported the use of qualifications (*n* = 130). Out of these samples, 103 (79.2%) specified U.S. workers, 10 (7.7%) specified an approval rating (e.g., above 95%), and 63 (48.5%) created a custom qualification (e.g., employed, having a supervisor, female, college educated, English speaking, Caucasian, owning a Facebook account, having access to a webcam, self-identified visible disability). Of the samples using qualifications, the majority reported using one (*n* = 92 or 70.77%) or two (*n* = 30 or 23.08%) qualifications.

##### Generalizability

Creating a qualification may influence generalizability in three ways. First, limiting a sample to a particular demographic (e.g., U.S. workers, employed workers, females) would limit generalizability to a broader demographic; however, this may increase validity if the research question is relevant to the demographic sampled (Highhouse and Gillespie, 2009). For example, researchers who are interested in studying organizational commitment will need to limit their sample to employed workers. Second, setting qualifications may indirectly impact the demographic makeup of the sample (Bates and Lanza, 2013). For example, U.S.- and India-based MTurk workers differ in their gender, education, and age composition (Ipeirotis, 2010; Ross et al., 2010). Finally, qualifications restricting participation to a high HIT approval rating, and/or a large number of HITs completed may increase the representation of Super Turkers in a sample.

##### Data quality

The accuracy and psychometric quality of MTurk data can be positively impacted by adding qualifications (Chandler et al., 2013). With respect to accuracy, workers meeting a HIT approval rate of 95% or above are more likely to pass attention checks and less likely to demonstrate social desirability compared to low reputation workers (Peer et al., 2014). Psychometric quality of the data likewise benefits from reputation qualifications, as specifying a HIT approval rate of 95% or above has been shown to increase internal consistency (Peer et al., 2014).

As mentioned earlier, limiting the location to the U.S. also tends to increase the psychometric quality of data (Litman et al., 2015). This may be due to language differences, as non-U.S. samples are less likely to speak English as a first language. Consequently, past research has found that non-U.S. samples do not demonstrate measurement equivalence of certain scales with U.S. samples (Steelman et al., 2014; Feitosa et al., 2015) and generally present other psychometric problems such as poor model fit (Steelman et al., 2014).

##### Recommendations

The qualifications specified will necessarily depend on the research question at hand. There is a balance between generalizability and validity in the type of qualification one specifies. Given the diversity of the MTurk sample, qualifications appear to be very important for evaluating the appropriateness of the final sample, as it changes the demographics of the sample. We recommend that all researchers using MTurk should report the qualifications used; justifications should also be provided when researchers decide on the types of qualifications to use (or lack thereof).

The choice of standard qualifications can enhance data quality. Foremost, for higher quality data, we recommend specifying a 95% approval rating or above to increase the chances of higher quality data (see Peer et al., 2014). The caveat is that specifying a 95% approval rating is not a sufficient condition for high quality data and other data screening methods (discussed later) should also be considered. Second, data will also likely be of higher quality when the language of the surveys match the first-language of the targeted region. By convention, limiting the location of the workers completing HITs to the U.S. is preferable unless attempting to conduct cross-cultural or country-specific research. From experience, participants may not accurately report their country location, and we recommend that researchers also check their IP addresses to confirm that they are located in the U.S. or use “first language” as a proxy. Finally, qualifications can increase the risk of non-naïveté, which may be a concern if one uses a fairly common experimental paradigm. We recommend specifying a smaller number of HITs approved (< 50 to100) to decrease the risk of non-naïveté.

Custom qualifications should be used with caution because workers may be dishonest, given that there is an incentive to qualify for a survey. As such, we recommend using prescreens (next section) rather than custom qualifications for selecting particular worker characteristics.

#### Prescreens

Prescreens are HITs (viz. a short survey/test before the actual study) used to indirectly screen out workers who are not in the desired target population (e.g., employed workers, males, minorities) or that do not exert sufficient effort (Bowling et al., 2016). There are three main ways to conduct prescreens. First, the main study HIT is set as hidden from MTurk workers but a prescreen HIT is posted separately and made viewable to MTurk workers; MTurk workers provide information to this prescreen HIT and workers with the desired characteristics are selected and invited to the main study HIT. For instance, a prescreen HIT may be a survey that asks workers to self-report their demographic characteristics (e.g., employment status, job title, tenure). Using the prescreen results, full-time employed workers are contacted by the requestor through their MTurk account for the main study HIT. A second way to screen workers is to embed the prescreen within a larger survey by using “branching” or “display logic” functions on survey platforms. For example, if the interest is to obtain full-time employed participants, employed workers can be directed to the study survey, and unemployed workers can be directed to a different survey (or to the end of the survey).

In our survey of the organizational research, 9 samples reported using prescreens out of 250 samples (3.6%). Prescreens have be used to select workers with specific work experiences (Burton et al., 2014; Yuan, 2014), health conditions (McGonagle and Hamblin, 2014), a certain level of ability (Effron et al., 2015), sufficient attention (Moore et al., 2015), or some combination of characteristics (Parker et al., 2015).

##### Generalizability

As with qualifications, prescreens *may* limit generalizability but may also increase validity when a research question calls for certain populations.

##### Data quality

From past research, it is difficult to know whether prescreens result in more complete data. However, we speculate that prescreens can be used to gauge motivation and attention, and thus screened workers may provide more complete data. Screening workers may also increase the accuracy of the data collected, particularly for tasks that are complex or require certain levels of knowledge or skill (Chandler et al., 2013). Additionally, the prescreen process is passive and may be less transparent, thus decreasing the likelihood of dishonest or socially desirable reporting (Shapiro et al., 2013).

##### Recommendations

Only a small percentage of studies reported using prescreens. We expect that for organizational research, selection through prescreens would likely be an important component to obtain relevant work samples. Thus, we recommend that researchers report any use of prescreens and provide the necessary information for reviewers and other researchers to evaluate the appropriateness of the prescreens. In addition, to understand the final sample composition, we propose that researchers should report on how many participants took the prescreen, how many participants qualified, and how many participants agreed to participate in the full survey.

Compared to qualifications, prescreens are a less direct way to sample certain populations or increase data quality. Researchers using a prescreen should be careful not to signal to workers what characteristics are desirable. Doing so increases the risk of dishonest or socially desirable responding. If screening for worker characteristics, we recommend not informing the worker that a prescreen is being used and including a variety of measures to keep workers from guessing the purpose of the prescreen. When screening for effort or ability, we recommend instructing workers to exert an appropriate amount of effort when completing the tasks, and where appropriate, separating the prescreen and main study in time to reduce fatigue. Researchers using this option should also carefully consider whether by screening for effort and ability, they may encounter range restriction (Hunter et al., 2006), or have decreased generalizability. When targeting a very specific and underrepresented population, it will be necessary to prescreen a large number of participants to find a large enough sample (e.g., McGonagle and Hamblin, 2014). Therefore, researchers are encouraged to increase the payment amount for the prescreen HIT in order to find a sufficient sized final sample.

We highly recommend finding ways to use a branching option to collect data on both the targeted and non-targeted population. Doing so allows for comparison of possible range restriction on the overlapping constructs. Further, the non-targeted population could be used to replicate using a different sample simultaneously. When using a branching option, workers in both the target and non-target population should be paid equivalent wages to avoid disgruntled workers who may post their complaints on message boards (Siegel et al., 2015). This reduces the number of workers who sign up for the HIT.

#### Self-selection

Like other convenience samples (Harber et al., 2003), self-selection will likely play a role in the final sample. Upon qualifying, MTurk workers can choose whether to work on the HIT resulting in self-selection into HITs based on payment, familiarity with the type of task, recruitment message, posts on message boards, and interest in the nature of the task. Even when workers start a HIT, they may also choose not to continue with it if the payment does not seem adequate for the time spent, if the task is too complex, or if the task is uninteresting. Self-selection (in and out of a HIT) is difficult to avoid (Horton et al., 2011), and may impact both generalizability and data quality.

Given that workers may be attracted to certain types of HITs, workers may participate in social science research HITs (as opposed to other types of HITs) or be more likely to participate in certain types of studies (Ross et al., 2010; Horton et al., 2011). At this juncture, it is difficult to know the extent of self-selection involved on MTurk. Ross et al. (2010) found that 52.9% of 573 workers reported completing surveys more often than other types of HITs; however, because they employed a survey methodology, we do not know if the reported percentages are based on a self-selected sample in the first place.

##### Generalizability

We do not currently have evidence one way or another that shows that self-selection impacts generalizability. In many ways, this issue reflects a broader concern of using convenience sampling where organizational or community respondents self-select to respond to surveys or studies (Landers and Behrend, 2015). One likely impact is that self-selection would lead to range restriction. Another likely impact is that workers who complete the study may have different characteristics as compared to those who have dropped out of the study.

##### Data quality

There is also limited research directly examining how self-selection impacts data quality. Understandably, completeness of data will suffer if MTurkers self-select out of the study. The extant research currently examines how factors such as payment can influence rate of data collection and quality of the data (Mason and Watts, 2010; Rogstadius et al., 2011; Crump et al., 2013; Litman et al., 2015). As will later be explicated, payment does not appear to impact data quality (e.g., internal consistency, accuracy) so much as the speed of data collection. Aside from examinations of payment, there is a lack of research examining how other factors (e.g., title of study, nature of task) influence self-selection and data quality.

##### Recommendations

Although a certain amount of self-selection is inevitable and perhaps unavoidable in MTurk samples, TurkPrime provides information (i.e., completion rate, bounce rate) that may indicate the extent of self-selection into and out of HITs (Litman et al., 2016). Specifically, the completion rate indicates how many participants dropped out of the study and the bounce rate indicates the percentage of workers who previewed a HIT and did not accept (start) the HIT. Low completion rates and high bounce rates may indicate the prevalence of self-selection. As Litman et al. also mentioned, researchers experiencing low completion or high bounce rates may want to consider the source of the problem (e.g., low payment, high cognitive load). We encourage researchers to use TurkPrime to track these issues of self-selection.

### MTurk as a work management system

MTurk can be viewed as a work management system, whereby researchers monitor, manage, and respond to work production to ensure quality work. The work management system is influenced by both controllable and less controllable factors. Controllable factors include compensation (i.e., payment, bonuses), work design (i.e., research design), and employing attention checks. Uncontrollable factors include the presence of MTurk message boards where workers discuss HITs.

#### Compensation

Prior to launching a HIT, researchers set a payment amount for a completed HIT. Work done on MTurk must be either automatically or manually approved by the researcher for the workers to be compensated. Generally, work completion is verified with a code found at the end of the study.

There are two types of compensation that may be awarded through Amazon: base pay and bonuses. Bonuses are optional, but can be awarded as an extra incentive, such as for higher quality work (Barger et al., 2011; Chandler et al., 2013). TurkPrime also makes awarding bonuses easier, as the program can be used to award bonuses to many workers simultaneously rather than one at a time as with the MTurk interface (Litman et al., 2016).

Our review substantiates the idea that collecting data on MTurk is less costly than other online research panels. In our survey of the organizational literature, a little under half (46.4%) reported a compensation amount. Compensation ranged from $0.10 to $5.00, with an average compensation amount of about $0.99 (*SD* = $0.93). Assuming roughly 30-min studies, this appears consistent with past research suggesting a payment of $0.75 for a 30-min survey (Barger et al., 2011). We can compare this to panels such as Qualtrics that charge ~$8 per participant for a 30 min survey (or even $32 for supervisors to fill in a 30 min survey) and Survey Monkey that has a maximum survey length of 30 min and a cost of about $3,000 for 500 participants, which comes out to be ~$6 per participant for a 30 min survey. Based on these quotes and other research comparing recruitment services (e.g., Brandon et al., 2014), MTurk participants are ostensibly paid at a lower cost than major survey companies. However, it is difficult to actually know whether participants themselves are paid at similar rates, as major survey companies do not reveal the actual rates paid to recruited participants and there are additional undisclosed overheads (e.g., sales, recruitment). Regardless, the end cost per participant for researchers is lower on MTurk compared to other online platforms.

##### Generalizability

There is little research examining the extent compensation amounts may affect generalizability. On the MTurk platform, HITs can be sorted by payment amount, thus it is easy for workers to self-select into HITs based on the compensation amount. It is possible that higher compensation amounts may attract Super Turkers or MTurkers who view MTurk as a substantial source of income. At the same time, higher compensation amounts may also attract more MTurk workers in general. Hence, we are unsure if compensation amounts would systematically affect generalizability.

##### Data quality

While the low payments allow research to be conducted at a low cost, there are concerns about how payment impacts the quality of the data on MTurk, and multiple researchers have investigated this matter. With regard to completeness of data, payment appears to influence attrition, as workers will weigh the length and complexity of the task against the amount of payment (Crump et al., 2013). It is likely that higher payment would lower attrition rates if MTurk workers perceive fair compensation.

With regard to accuracy, several researchers have found the financial incentives do not increase the accuracy of the data collected on MTurk (Mason and Watts, 2010; Rogstadius et al., 2011; Crump et al., 2013). Likewise, payment does not generally appear to influence the psychometric quality of the data in the form of internal consistency (Litman et al., 2015). Litman et al. (2015) examined data quality in relation to compensation in both India-based workers and U.S.-based workers, and found that while internal consistency was not significantly impacted by payment for U.S.-based workers, internal consistency estimates increased when offering above minimum wage in India, but decreased when offering much higher than minimum wage. Critically however, these internal consistency differences were very small and may be simply a result of sampling variability. Overall, this suggests that low payments do not necessarily result in poor or inaccurate data.

##### Recommendations

Compensation is of paramount importance to MTurk workers, as compensation is a main motivator for completing work on MTurk (Paolacci et al., 2010; Behrend et al., 2011; Kaufmann et al., 2011). While the amount of compensation does not appear to be affected by data quality, we urge researchers to make ethical considerations when determining payment (Bederson and Quinn, 2011; Fort et al., 2011; Bergvall-Kåreborn and Howcroft, 2014). There are benefits to providing higher compensation as it is related to greater speed and quantity of data collection; likely because workers can sort HITs by level of payment (Mason and Watts, 2010; Faridani et al., 2011; Rogstadius et al., 2011). Higher compensation is also associated with lower attrition rates (Crump et al., 2013). Moreover, very low rates of compensation can lead to worker dissatisfaction (Brawley and Pury, 2016) and this may be conveyed to other MTurk workers, lowering the number of HITs completed. There is a strong movement in the Turker community to shame requesters who pay less than the national minimum wage. This can take the form of emails to the requester or posting messages on public forums such as Turkopticon that identify “unfair” employers. From experience, we suggest making terms of payment *very* clear to participants on the outset to avoid problems. Researchers should base payment on the nature of the task, the time it tasks to complete the task, and the cognitive load.

We also recommend that researchers provide compensation quickly. Researchers should avoid delaying payment to avoid emails from disgruntled workers and negative reviews on message boards. Using the automatic approval mechanisms on TurkPrime is one way to avoid delayed payment (Litman et al., 2016). TurkPrime allows for the option of automatically accepting work that is given either a fixed code or dynamic code (programmed through a survey creation system). Any work that does not provide the correct code requires researcher approval (Litman et al., 2016). Consistent with Litman et al. (2016), we recommend using a dynamic code that is unique for each worker to avoid the potential for the code being shared on message boards.

At this point, less than half of the organizational studies using MTurk reported compensation amount. In an effort to promote transparency and allow for future meta-analyses examining the effect of payment on sample characteristics, we recommend reporting the payment amount given to participants and whether any bonuses were offered. We also suggest including the average task time or including the hourly rate of participation.

#### Research design

When considering collecting data on the MTurk platform, the appropriateness of the research design is an important factor. There are numerous examples in the organizational literature of non-experimental (e.g., Cho and Allen, 2012; Wiltermuth and Flynn, 2013; Long and Christian, 2015), experimental (e.g., Chua, 2013; Casciaro et al., 2014; Huang et al., 2015a; Inesi and Cable, 2015; Welsh et al., 2015), and time-separated designs (e.g., Wiltermuth et al., 2013; Parker et al., 2015) being implemented on MTurk. Out of the 250 MTurk samples included in the current review, 173 samples (69.2%) used an experimental design; 52 (20.8%) used a correlational design; 19 samples (7.6%) were used for scale development; and 6 samples (2.4%) used a time-separated design. This suggests a preference for organizational researchers, or at least those in the top organizational journals, to use MTurk for online experimental studies where many criticisms about MTurk as a research platform (e.g. the ability to lie, inattention on surveys) may be less of a concern given random assignment.

In our survey of the organizational literature utilizing MTurk, the experimental manipulations used on MTurk ranged broadly from avatar video-based studies (Marchiondo et al., 2015), to vignette studies (Kovács et al., 2014), to policy capturing (Young, 2016). Likewise, the variables of interest varied widely. To examine the topics covered using MTurk samples, we coded the articles based on the 26 content areas specified by the Society for Industrial and Organizational Psychology, Inc. in the 2016 *Guidelines for Education and Training in Industrial-Organizational Psychology*. The content areas specified in the guidelines provide a useful tool for examining how researchers are using MTurk. As can be seen in Table 4,^1^, the topics examined using MTurk samples varies widely. The most common examinations involve the study of attitudes (*n* = 41), followed by judgment and decision-making topics (*n* = 30) and ethical, legal, diversity, and international issues (*n* = 26). The high representation of these three topics should not be surprising. As previously stated, around 20.8% of the MTurk samples used a correlational design making attitudinal research a popular option on MTurk. Likewise, another 69.2% of the samples used an experimental design making judgment and decision-making research a viable option. Finally, MTurk samples are more diverse than other samples creating opportunity for researchers interested in cultural issues or diversity more generally.

**Table 4:** Topics examined in studies using MTurk participants.

**Topics^a^**	**Number of articles**
Ethical, legal, diversity, and international issues	26
Fields of psychology	–
History and systems of psychology	–
Professional skills	–
Research methods	9
Statistical methods/data analysis	2
Attitude theory, measurement, and change	41
Career development	10
Criterion theory and development	–
Groups and teams	3
Human performance	9
Individual assessment	–
Individual differences	13
Job evaluation and compensation	1
Job/task/work analysis, competency modeling, and classification	–
Judgment and decision-making	30
Leadership and management	18
Occupational health and safety	7
Organizational development	–
Organization theory	–
Performance appraisal/management	2
Personnel recruitment, selection, and placement	6
Training, theory, delivery, program design, and evaluation	1
Work motivation	1
Consumer behavior	–
Human factors	–

a *Society for Industrial Organizational Psychology Inc. (2016)*.

*Articles may contain more than one topic area*.

Many MTurk samples were used as pilot tests (e.g., Lin-Healy and Small, 2012; Burton et al., 2014; Nichols and Cottrell, 2014; Spisak et al., 2014; Bhargave et al., 2015; Rosette et al., 2015; Shirako et al., 2015; van Dijke et al., 2015) or for further psychometric work (e.g., McGonagle and Hamblin, 2014; Howell et al., 2015; McGonagle et al., 2015; Milkman et al., 2015; Rosette et al., 2015; Walter et al., 2015). Some of these samples were included as a footnote or in an Appendix to act as a supplement to the main study.

The literature shows that MTurk can be leveraged for a wide variety research designs. However, researchers need to be aware of potential opportunities and challenges posed by experimental and non-experimental (cross-sectional and longitudinal) designs. We discuss how research designs may be related to the issues of generalizability and data quality.

##### Generalizability

There has been little research examining how the different types of research designs may impact generalizability given the types of MTurk workers who may be most attracted to such studies. However, there is some indirect evidence that can be gleaned on this issue depending on the type of research design. We have discussed issues pertaining to non-experimental cross-sectional research in previous sections, thus we focus on experimental and longitudinal designs here. With regard to experimental designs, past research examining the MTurk platform has generally replicated various common experimental paradigms and effects (e.g., prisoner's dilemma, anchoring) (Paolacci et al., 2010; Horton et al., 2011; Amir et al., 2012; Berinsky et al., 2012; Crump et al., 2013; Summerville and Chartier, 2013; Wolfson and Bartkus, 2013; Bui et al., 2015). This suggests that, at least with well-established paradigms, these are replicable in an MTurk sample, suggesting a level of generalizability. At this point, less is known concerning whether less established paradigms and phenomena will replicate. The key for organizational researchers is that experimentation on MTurk is possible and encouraged (Highhouse and Zhang, 2015).

With regard to longitudinal designs, features of the design will likely affect the final composition of the sample and the generalizability. Past research suggests that individuals responding to surveys at the different time points may differ in both demographic and other characteristics. Daly and Nataraajan (2015) found that participants who responded to a second or third wave of data collection were significantly older, had significantly more education, were higher on conscientiousness and agreeableness, and were more likely to be female. Notably, the time between surveys may also have an impact, and the differences may be greater with more waves of data collection or longer time periods between waves of data collection (Daly and Nataraajan, 2015). At the same time, we note that these issues that have been identified are not unique to MTurk samples alone (Goodman and Blum, 1996).

##### Data quality

We focus on data quality of experimental and longitudinal research here as past sections on data quality can be applied to non-experimental cross-sectional research. With regard to experimental research, MTurk samples are at risk of worker non-naïveté and selective attrition as a function of experimental condition posing a risk for both completeness and the accuracy of inferences drawn (Chandler et al., 2014; Zhou and Fishbach, 2016). On the other hand, MTurk is less likely to fall prey to experimenter effects and the online platform offers an easy means of random assignment and standardized procedures (Highhouse and Zhang, 2015). Therefore, there appears to be a balance that needs to be weighed by researchers.

A main concern with longitudinal research conducted on MTurk is whether data are complete; that is, whether retention (or conversely, attrition) rates are higher. According to the MTurk literature (Buhrmester et al., 2011; Shapiro et al., 2013; Schleider and Weisz, 2015), the completion rates in MTurk studies have an average of 62.72% for an initial follow-up and 79.45% after the initial follow-up. Importantly, retention rates depend on the length of time between survey administrations. We found that there was an 80% retention rate for surveys weeks apart (Shapiro et al., 2013), and a 75% retention rate 2 months apart (Daly and Nataraajan, 2015), and a retention rate of 47% after 13 months (Daly and Nataraajan, 2015). Critically, the 47% retention rate compared to nationally representative annual surveys is low (around 90%; Gustavson et al., 2012; Schoeni et al., 2013), although large scale epidemiological surveys have completion (attrition) rates that fall within the 30 to 70% range. With regard to daily dairy studies, previous research found a 60% retention rate over 14 consecutive days (Boynton and Richman, 2014). This was slightly lower than typical rates found in college samples (75–85%). In general, lower rates can affect data quality in different ways including systematically losing respondents of specific attributes.

##### Recommendations

As previously mentioned, there may be different considerations when utilizing non-experimental cross-sectional, experimental, and longitudinal research with MTurk workers. Across the different designs, inattention and careless responding is a concern. Possible solutions include monitoring attention through including checks (to be described later) (Meade and Craig, 2012), monitoring the time taken to complete the study, shortening the length of the survey to decrease the risk of fatigue (Fleischer et al., 2015), and having clear instructions and items (Alonso and Mizzaro, 2012). Given that extant online surveys record the time taken for completion, a simple proxy for attention and accuracy is the time taken to complete the survey; a very short time taken would be indicative of inattention and inaccuracy.

In experimental designs, we view selective attrition to be a real threat to internal validity. Specifically, attrition may be a function of the experimental manipulations (Rand, 2012; Crump et al., 2013; Zhou and Fishbach, 2016). Along with Zhou and Fishbach (2016), we propose several steps researchers can take to address attrition. First, prescreens can be conducted to identify less motivated participants and exclude them from the experimental study. Second, we encourage researchers to do initial pilot tests on MTurk and to determine if there are differential attrition rates between conditions. Third, given the non-negligible rates of attrition, we encourage researchers to report and analyze attrition across different conditions. We encourage researchers to use TurkPrime as it provides information about the completion rate in real time.

To control for non-naïveté in experimental designs, researchers should also attempt to screen out (or control for) participants who have participated in similar experiments (Paolacci et al., 2010) and avoid common research paradigms when possible (Chandler et al., 2014). Some research suggests that individuals most familiar with survey research are the least likely to read instructions carefully (Hardy and Ford, 2014); therefore, instructional manipulations (i.e., manipulations that give different instructions to different participants) need to be undertaken with care. If the experimental design employs an instructional manipulation, the researcher should be sure to conduct an instructional manipulation check (as it is also best practice to do so).

In cross-sectional non-experimental and experimental designs, we recommend using TurkPrime to create smaller batches that can be programmed to release smaller numbers of HITs on different days of the week and different times of the day (Litman et al., 2016). Using smaller batches increases the likelihood that the HITs will be completed by individuals who have different MTurk habits (e.g., completing at work vs. after work), will increase the speed of data collection because the HITs are sorted newest to oldest by default, and will allow researchers to monitor data collection.

In longitudinal designs, researchers should be aware of the potential for lower retention rates (Chandler et al., 2014) and whether low retention may bias the sample (Goodman and Blum, 1996). We encourage researchers to consider past studies reviewed here as a benchmark for retention rates over different time frames to determine the size of the initial sample for an appropriate final sample size. In general, attrition rates depend on the level of incentives researchers have to encourage continued participation. We encourage researchers to increase the amount of compensation when the follow-up time frame increases to improve retention rates.

We recommend the use of TurkPrime for longitudinal designs in MTurk. TurkPrime makes time-separated data collection easier through the use of worker groups, batch email features, and by matching worker IDs across multiple data collections when using survey creation software (Litman et al., 2016). In particular, the batch email feature allows researchers to contact all eligible workers (such as those in a desired worker group) simultaneously as opposed to one at a time on the MTurk platform. Additionally, the email notification can include information about the study and automatically includes the study link.

#### Attention checks

Attention checks are implemented to screen for careless or insufficient-effort responding. Careless or insufficient-effort responding is a serious issue for sampling in general (Meade and Craig, 2012; Huang et al., 2015b; Kam and Meyer, 2015; Ran et al., 2015), but particularly for MTurk samples because many workers are likely incentivized to complete HITs quickly to maximize their return on investment (Barger et al., 2011). DeSimone et al. (2015) identified three types of data screening methods: direct, archival, and statistical. Direct techniques include self-report indices (e.g., “I did not pay much attention to this survey”), instructed items (e.g., “If you are paying attention select strongly disagree for this item”), and bogus items (e.g., “All my friends are mermaids”). Archival techniques include identifying semantic synonyms and antonyms, monitoring response times, or identifying long-string or invariant responding (e.g., straight-lining). Statistical techniques include psychometric synonyms and antonyms (i.e., using inter-item correlation cutoff scores), personal reliability (i.e., response consistency), and multivariate outlier analysis.

We examined the MTurk samples in the organizational journals surveyed and found that only 22.8% of samples reported the use of attention checks (*n* = 57). Out of the samples reporting an attention check, the majority used direct techniques for detecting inattention: 25 (43.9%) included at least one instructed item, 1 (1.8%) included at least one bogus item, 9 (15.8%) asked the worker to recall information from instructions or an article, and 1 (1.8%) directly asked workers if they were paying attention. 12.28% of the attention checks were archival: 6 (10.53%) monitored the time spent on the task and 1 (1.8%) examined the data for long-string or invariant responding. The remaining 17 samples had unspecified attention checks. Thus, no samples reported using statistical methods for detecting insufficient effort responding. Only two samples (Burton et al., 2014; McGonagle et al., 2015) reported using more than one attention check in the study, and both utilized instructed items and monitored time spent. On average, attention checks resulted in ~43 workers (*SD* = 70) being excluded, which equates to roughly 13% of the sample, on average.

##### Generalizability

The use of attention checks impacts both the size and makeup of samples; that is, the choice to exclude individuals based on failed attention checks will result in a smaller sample size, and may alter the composition of the sample itself. Research on MTurk has found that women, older adults, professionals, and students are more likely to answer attention check questions correctly (Downs et al., 2010). Likewise, U.S. participants are more likely to correctly answer attention check questions compared to non-U.S. participants (Goodman et al., 2012). Thus, including attention checks may decrease generalizability due to smaller sample sizes and sampling individuals who are less likely to be male, from different cultures, and younger (Berinsky et al., 2016). Taken together, the use of attention checks may limit the generalizability of the sample although it will increase the data quality from which one draws inferences.

##### Data quality

The use of attention checks should have a positive impact on data quality. As many commentators point out, careless or inattentive responding results in error variance (or noise; Goodman et al., 2012; Fleischer et al., 2015; Huang et al., 2015b; McGonagle, 2015; Ran et al., 2015; Rouse, 2015). Thus, attention checks increase the accuracy of responses and increase psychometric quality (e.g., internal consistency) of the data by monitoring careless or insufficient effort responding. Importantly, research has also shown that MTurk samples pass attention checks at similar rates as other samples (Paolacci et al., 2010; Goodman et al., 2012), indicating that MTurk data quality is similar based on attentiveness.

##### Recommendations

There are many recommendations on screening data for inattention. We recommend referring to DeSimone et al. (2015) for best practices and recommendations during study design, administration, and cleaning, as well as Huang et al. (2015a) and Meade and Craig (2012). Below we make a few specific recommendations based on this literature, as well as, the MTurk literature. Specifically, we recommend using multiple ways to detect suspect responses (Kittur et al., 2008; DeSimone et al., 2015), considering the appropriateness of data cleaning procedures for a given research design (Harms and DeSimone, 2016), considering how attention checks will influence compensation, and deciding a priori how the data will be screened using attention checks.

Past research has questioned the validity of using *a single* attention check to detect inattention (Chandler et al., 2013; DeSimone et al., 2015; Harms and DeSimone, 2015). Downs et al. (2010) found that 88% of the MTurk workers were able to answer the easy attention check question correctly, whereas, only 64% were able to answer the difficult attention check question correctly. Taken together, attention checks are not all created equal and some may be more effective than others. Indeed, DeSimone et al. (2015) pointed out that certain attention check methods are better suited to certain types of responses (random, invariant, acquiescent, extreme). Therefore, we recommend that researchers consider the use of several different types of attention checks.

Researchers will also need to decide whether failure on attention checks should lead to withholding compensation. We recommend that, if possible, researchers provide payments to workers regardless of whether they pass attention checks to avoid disgruntled workers and message board posts that may inadvertently affect the HIT response. Analyzing whether MTurk workers pass attention checks in real time is time consuming and may be impractical for timely compensation. Additionally, attention checks can serve the purpose of screening data without serving the purpose of deciding payment. If researchers withhold compensation based on attention check failures, this should be explicitly mentioned in the instructions so that MTurk workers experience procedural fairness.

How attention checks are used to screen data should be transparent in reporting and decided a priori. One concern raised by researchers is overzealous *post-hoc* data cleaning (Chandler et al., 2014; Paolacci and Chandler, 2014; Harms and DeSimone, 2016), which may lead to problems in inferences. We highly recommend transparent reporting of how many participants were collected from MTurk, the nature of any attention checks, how attention checks were implemented, any exclusion criteria, and how many participants were removed from the final analysis.

#### Message boards

There has been limited research on how message boards impact the generalizability and quality of the data collected on MTurk. Participants on MTurk have the ability to post and view message boards (e.g., turkopticon, reddit, mturkgrind, turkernation) that may contain information about the nature of the HIT, the presence and nature of data-quality check questions, payment, and so forth (Mason and Suri, 2012). However, the typical message board post focuses on interesting HITs, bad requestors, or high paying HITs.

##### Generalizability

Message boards have the ability to direct traffic to a HIT, and thus, impact self-selection into HITs (Chandler et al., 2014). Additionally, the population of workers using message boards may not be representative of MTurk as a whole (Chandler et al., 2014), and are more likely to be a sample of MTurk workers with some weak social ties (Schmidt, 2015) or Super Turkers. Taken together, message board activity may result in a sample of MTurk workers that differs from the MTurk population in meaningful ways.

##### Data quality

There is currently no research examining message boards as a threat to data quality. However, due to the risk of non-naïveté (Chandler et al., 2014), we speculate that usefulness of prescreens, attention checks, or experimental designs may be adversely impacted.

##### Recommendations

Although there is no way to prevent participants from posting or viewing these message boards, researchers do have the ability to monitor the boards. We recommend that researchers monitor message boards to determine if compromising information has been shared (e.g., prescreen criteria). Another use of monitoring message boards is to track if negative information about the study has been put online. This could substantially lower the response rates on a research HIT. In addition, if researchers are concerned that compromising information may be shared on the message boards eventually, they should consider releasing the research HIT in one large batch to alleviate time for online discussion (Horton et al., 2011).

## Discussion

The rise in popularity of the MTurk platform in the organizational sciences does not appear to be abating. At the same time, there are many questions and concerns that have been raised about using MTurk as a platform for data collection. To address these questions and concerns, we have conducted, in our view, the most integrative review to date, along with providing specific recommendations on the use of the MTurk platform, which will benefit organizational researchers and social scientists in general. Based on our review and recommendations, we provide a detailed reporting checklist in Table 5. We conclude by discussing the proposed systems framework and future research directions.

**Table 5:** Reporting checklist for MTurk samples.

**Reporting checklist**	
Worker characteristics	Sample Size Average age Gender (e.g., %Male) Education Percentage from U.S. Racial/Ethnic makeup First language Employment status (full-time, part-time, unemployed)
Qualifications	Nature of qualifications
Prescreens	Nature of prescreen Implementation of prescreen % Excluded based on prescreen Number of participants agreeing to participate in main study
Self-selection	Bounce rate (when possible) Completion rate (when possible)
Compensation	Base payment Bonuses Average task time
Research design	Nature of research design Materials used Manipulations Number of participants in each condition (experimental design) Attrition rates/Attrition rates per condition (experimental design)/Attrition rates between waves of data collection (time-separated design) Time between waves of data collection (time-separated design)
Attention checks	Nature of attention checks Number of participants removed from final analysis

### Systems framework

**Figure 2:**
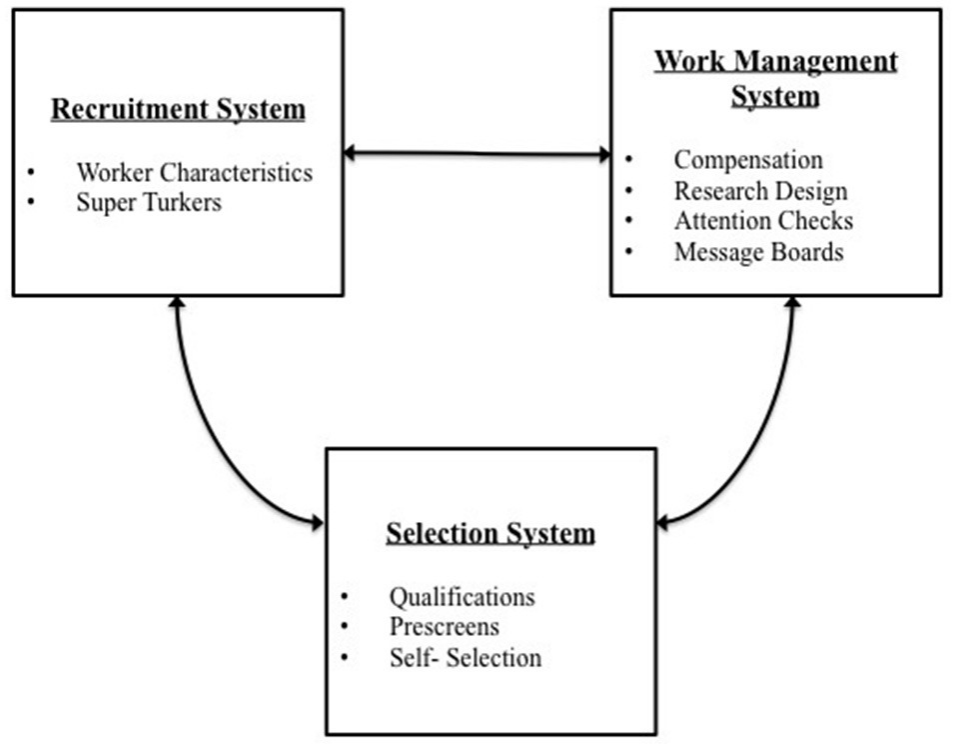
Systems perspective of MTurk.

Each system in the framework that we have provided for organizing and evaluating the MTurk platform was presented in a sequential manner. However, these systems do not work in isolation to influence data quality and generalizability. Rather, factors making up the recruitment, selection, and work management systems influence factors in other systems (see Figure 2). The use of qualifications will influence worker characteristics, Super Turkers, and self-selection into HITs. On the other hand, prescreens and attention checks can also influence worker characteristics and may have a reciprocal relationship with MTurk message boards if workers are forewarned about attention checks or prescreens. Further, compensation can influence worker characteristics and self-selection into HITs. The type of research design can also influence self-selection into HITs and message board participation (e.g., “this task was interesting”).

Apart from the practical contribution to MTurk usage, a conceptual contribution of this systems framework is that it can be used in the broader discussion of data collection and convenience sampling. Many different sampling methods can be viewed as comprising a recruitment system (population from whom sample is recruited), a selection system (factors involved in selecting participants), and a work management system (factors involved in monitoring and managing the research). The individual factors comprising these systems will vary; however, the overarching systems remain the same. We propose that researchers can consider other types of data collection modes (e.g., online panel data, psychology subject pool) within this frame of reference to consider the appropriateness of a given sample for the research questions at hand.

### Future research directions

As we note in many sections, there is a lack of research examining how various factors influence generalizability and data quality. We urge researchers to consider the areas noted in Supplementary Tables 1–3 to further examine areas that do not have the best practices presented due to a lack of research. Over time, consolidated research on both procedures for collecting data on MTurk (e.g., qualifications, payment), representativeness (e.g., sample characteristics), and quality of the data (e.g., completeness, accuracy, and psychometric quality) will shed more light on the efficacy of MTurk samples for organizational research.

## Conclusion

The aim of the current article was to provide organizational researchers with an integrative resource with which to judge the appropriateness of MTurk samples for use in their research. We organized past research examining and utilizing MTurk into an organizing framework that views MTurk through a systems perspective. Our general conclusion is that MTurk provides researchers with a unique opportunity to sample a wider portion of the working population than traditional student or community samples and in a quick and cost effective way. With opportunities, however, come challenges unique to MTurk. We view MTurk as another means of data collection and we urge researchers to consider their research design and research questions. We hope that this article presents the information necessary for organizational researchers (and researchers in related fields) to make educated decisions about whether or not to utilize MTurk and how best to do so. We additionally hope that the current review will stimulate future research to fill current gaps in our understanding of how MTurk impacts our science.

## Author contributions

MK: Wrote the paper, conducted literature search, and coded the studies when necessary. LT and PH: Helped with the framing of the paper, writing, and editing.

### Conflict of interest statement

The authors declare that the research was conducted in the absence of any commercial or financial relationships that could be construed as a potential conflict of interest.

## Footnotes

^1^We also provide information about the specific variables examined and how these variables were coded in the Supplementary Materials (Supplementary Table 4).
