# Supplementary material for: Systems Perspective of Amazon Mechanical Turk for Organizational Research: Review and Recommendations
Source: Front Psychol. 2017 Aug 8;8:1359. doi: 10.3389/fpsyg.2017.01359 (PMC5550837; doi:10.3389/fpsyg.2017.01359)
Supplement: Supplementary file 1 [file Table1.PDF]

Supplementary Materials

Supplementary Table 1

MTurk as a Recruitment System

	Generalizability	Data Quality	Recommendations
**Worker Characteristics**	• MTurk samples are more diverse in terms of education and age than college samples.<br>• MTurk samples are younger and more educated than community samples; however, these differences may not be statistically significant.<br>• MTurk samples are most similar to other Internet samples.<br>• MTurk samples are not nationally representative, and should not be treated as such.<br>• MTurk samples contain both employed and unemployed workers.<br>• MTurk workers are employed in a wide variety of industries.<br>• MTurk samples may report higher levels of negative constructs and lower levels of positive constructs compared to other samples; however, this requires further empirical examination.	**Completeness**<br>• MTurk samples are less likely to complete surveys compared to student (lab) samples.<br>• Dropout rates are similar for surveys conducted online and MTurk samples.<br><br>**Accuracy**<br>• MTurk samples may be prone to inattention due to lack of environmental control, social desirability bias, and dishonesty.<br>• MTurk participants have been shown to be more attentive to instructions than undergraduate participants.<br><br>**Psychometrics**<br>• MTurk samples have demonstrated acceptable internal consistency, test-retest reliability, interrater reliability with the MTurk sample, and interrater reliability with experts.<br>• MTurk samples have demonstrated measurement equivalence with student and employee samples.<br>• MTurk samples from non-Native English speaking countries do not fair as well psychometrically.	• Consider whether MTurk participants are appropriate for the target population.<br>• Conduct research on issues surrounding range restriction in MTurk samples.<br>• Be aware of higher dropout rates in MTurk samples.<br>• Use caution when sampling from an MTurk population with a large number of non-English speakers.

	Generalizability	Data Quality	Recommendations
**“Super Turkers” (professional test takers)**	• Researchers may be sampling from a small pool of workers who are completing the majority of the HITs presenting a problem for sample independence.<br>• Super Turkers may view MTurk as a primary job, and may view work in significantly different ways.	**Completeness**<br>• There is currently no evidence to suggest that Super Turkers impact the completeness of data collected on MTurk.<br><br>**Accuracy**<br>• Repeated participation in experiments may impact the effectiveness of the manipulations due to learning and practice effects and inferring demand characteristics.<br>• Measures of ability may be inflated due to practice effects among Super Turkers.<br><br>**Psychometrics**<br>• There is currently no evidence to suggest that Super Turkers impact the psychometric quality of data collected on MTurk.	• Recognize the active population of MTurk workers is much smaller than the 500,000+ workers registered on MTurk.<br>• Prevent repeated participation across studies by assigning qualifications to each worker ID, monitoring work IDs across studies, or by using TurkPrime.<br>• Consider the risk of non-naïveté given the nature of the research design.<br>• When employed individuals are the target population, include items requesting a specific job title, occupation, etc.<br>• Use caution when seeking to understand job-related issues from MTurk respondents.
