# Supplementary material for: Systems Perspective of Amazon Mechanical Turk for Organizational Research: Review and Recommendations
Source: Front Psychol. 2017 Aug 8;8:1359. doi: 10.3389/fpsyg.2017.01359 (PMC5550837; doi:10.3389/fpsyg.2017.01359)
Supplement: Supplementary file 2 [file Table2.PDF]

Supplementary Table 2

MTurk as a Selection System

	Generalizability	Data Quality	Recommendations
**Qualifications**	• Limiting MTurk samples to a particular demographic limits generalizability to a broader demographic.<br>• Qualifications (e.g., location) may impact the characteristics (e.g., gender, education, age) of a sample.<br>• Specifying Master Turkers, requesting high HIT approval rates (high reputation), and/or a high number of HITs approved (high productivity) may increase the likelihood of sampling from the pool of workers who view MTurk as a job (i.e., Super Turkers).	**Completeness**<br>• Qualifications are not likely to impact the completeness of data collected on MTurk.<br><br>**Accuracy**<br>• High reputation workers (i.e., approval rating of 95% or above) have been shown to increase the likelihood of passing attention check questions and decrease social desirability.<br><br>**Psychometrics**<br>• High reputation workers increase internal consistency.<br>• Requesting U.S. (or English-speaking countries) results in favorable measurement equivalence, higher internal consistency, and favorable model fit indices.	• Consider the research question at hand.<br>• Use 95% approval rating to increase the quality of the data.<br>• Limit the location of HITs completed to the U.S. unless conducting cross-cultural or country-specific research.<br>• As workers may not accurately report their location, it is important to also check IP addresses or use “first language” as a proxy.<br>• Use lower previous HIT approvals when using common experimental paradigms; however, use caution as these samples may result in lower data quality and/or be less representative of the general MTurk population.<br>• Use custom qualifications with caution, as workers are not always honest. Consider using prescreens in place of custom qualifications where possible.<br>• Report all qualifications used, and provide justification for the presence or absence of qualifications.

	Generalizability	Data Quality	Recommendations
**Prescreens**	• May be used to target specific worker characteristics (e.g., gender, employment), thus reducing overall generalizability of working population, but may be useful for studying specific subpopulations.	**Completeness**<br>• Prescreens for gauging motivation may provide more complete data.<br><br>**Accuracy**<br>• Prescreens can increase the quality of data collected for tasks that are complex or require a certain level of knowledge or skill.<br>• More passive than qualifications, decreasing the risk of dishonest or socially desirable reporting.<br><br>**Psychometrics**<br>• Prescreens have not been shown to impact the psychometric quality of data collected on MTurk.	• Use prescreens to obtain relevant work samples.<br>• Use prescreens to indirectly sample certain populations or increase data quality.<br>• When selecting for sample characteristics, avoid signaling workers to the desired characteristics.<br>• When screening for effort or ability, inform workers that their effort or ability is important.<br>• Weigh the risk of reducing generalizability and the risk of range restriction when screening for effort and ability.<br>• Use a branching option to collect data on both targeted and non-targeted populations to allow for examination of range restriction on overlapping constructs and for simultaneous replications with different populations.<br>• Target populations and non-target populations should be paid equivalently.<br>• Report whether prescreens were used, implemented, and how many participants did not qualify, qualified, and how many agreed to participate in the main study.

	Generalizability	Data Quality	Recommendations
**Self-Selection**	• Limited research that suggests self-selection impacts generalizability.<br>• Self-selection may impact range restriction.	**Completeness**<br>• Self-selection out of HITs results in missing or incomplete data. Factors such as length of study, cognitive demand, and payment are likely to effect such self-selection.<br><br>**Accuracy**<br>• Self-selection is not likely to impact accuracy of data collected on MTurk.<br><br>**Psychometrics**<br>• There is a lack of empirical evidence suggesting that self-selection impacts the psychometric quality of data collected on MTurk.	• Consider using TurkPrime to gather information about completion and bounce rates, which indicate the degree of self-selection out of HITs and into HITs, respectively.<br>• Consider how factors such as compensation and research design interact to influence self-selection.<br>• Conduct research on how study characteristics influence self-selection.
