# Supplementary material for: Systems Perspective of Amazon Mechanical Turk for Organizational Research: Review and Recommendations
Source: Front Psychol. 2017 Aug 8;8:1359. doi: 10.3389/fpsyg.2017.01359 (PMC5550837; doi:10.3389/fpsyg.2017.01359)
Supplement: Supplementary file 3 [file Table3.PDF]

Supplementary Table 3

MTurk as a Work Management System

	Generalizability	Data Quality	Recommendations
**Compensation**	• Payment may impact self-selection into HITs.<br>• Do not have empirical evidence that compensation impacts generalizability.	**Completeness**<br>• Compensation is likely to influence the speed of data collection and attrition.<br><br>**Accuracy**<br>• Payment does not relate to output accuracy.<br><br>**Psychometrics**<br>• Payment does not generally impact internal consistency, but may impact internal consistency for Indian workers.	• Payment should be ethical, and determined based on the nature of the task, the time it takes to complete the task, and the cognitive load.<br>• Make terms of payment clear to MTurk workers.<br>• Avoid delaying payment for both ethical and practical reasons.<br>• Use a dynamic code for each worker that can be automatically accepted through TurkPrime.<br>• Report base pay, bonuses, and average task time.

	Generalizability	Data Quality	Recommendations
**Research Design**	• Based on replication studies, MTurk appears to be viable for experimental research.<br>• In longitudinal designs, the sample characteristics may differ in subsequent waves of data collection due to attrition.	**Completeness**<br>• Experimental designs are at risk for selective attrition as a function of experimental condition.<br>• Longitudinal designs are possible on MTurk, although attrition may be higher than other samples. Attrition rates will be influenced by time between surveys and incentives.<br><br>**Accuracy**<br>• Worker non-naïveté poses a risk to the accuracy of inferences drawn from experimental designs.<br>• MTurk samples are less likely to exhibit experimenter effects.<br>• MTurk also permits standardized procedures and random assignment for experimental designs.<br><br>**Psychometrics**<br>• Psychometric quality of the data will be a function of study measures.	• Across all research designs, monitor attention, monitor time spent completing the study, and have clearly worded instructions and items.<br>• Manage selective attrition by prompting less motivated participants to drop out prior to the manipulation, conduct a pilot study to test for selective attrition, and report attrition across conditions.<br>• Avoid common experimental paradigms when possible and attempt to screen out (or control for) participants who have participated in similar experiments.<br>• Use instructional manipulation checks when using manipulations reliant upon such cues.<br>• Use TurkPrime to release smaller batches of HITs to sample MTurk workers who may have different schedules for completing HITs.<br>• When conducting longitudinal research, collect a larger initial sample and monitor attrition rates.<br>• Time separated research should consider the appropriate compensation and time between waves of data collection.<br>• Use TurkPrime for longitudinal data collection.

	Generalizability	Data Quality	Recommendations
**Attention Checks**	• Use of attention checks may result in a sample that is less likely to be male, from different cultures, and younger.	**Completeness**<br>• Attention checks do not appear to impact the amount of missing data or attrition.<br><br>**Accuracy & Psychometrics**<br>• Attention checks increase data quality by reducing error variance (i.e., "noise") resulting in more accurate inferences and data that is more psychometrically sound.<br>• MTurk participants have been found to pass attention checks at similar rates as other samples.	• Use multiple methods for detecting inattention.<br>• Consider the appropriateness of data cleaning procedures for a given research design and the likelihood of various types of response bias.<br>• If possible, pay workers regardless of whether they pass attention checks to avoid disgruntled workers.<br>• If withholding compensation based on attention check failures, this information should be disclosed to MTurk workers prior to the study.<br>• Decide a priori how data will be screened.<br>• Report any use of attention checks, the nature of these attention checks, and how many participants were removed from the final analysis.
**Message Boards**	• Message boards may impact selection into HITs.<br>• Workers using the message boards may differ from the general population on MTurk, may be more likely to be Super Turkers, and are more likely to have weak social ties with other MTurk workers.	**Completeness**<br>• Message boards are unlikely to impact the completeness of data.<br><br>**Accuracy**<br>• May negatively impact the usefulness of prescreens, attention checks, and experimental designs.<br><br>**Psychometrics**<br>• Message boards are unlikely to impact psychometric data quality.	• Monitor message boards for compromising or negative information.<br>• Consider releasing one large batch to reduce time for online discussion.
