# Supplementary material for: Systems Perspective of Amazon Mechanical Turk for Organizational Research: Review and Recommendations
Source: Front Psychol. 2017 Aug 8;8:1359. doi: 10.3389/fpsyg.2017.01359 (PMC5550837; doi:10.3389/fpsyg.2017.01359)
Supplement: Supplementary file 4 [file Table4.docx]

**Supplementary Materials**

Supplementary Table 4

| Citation | Journal | Variables | Topic Categorization |
| --- | --- | --- | --- |
| Adam et al. (2015) | *OBHDP* | - Self-construal - Location of self - Money donated to charity - Judgment of controversial medical issues | - Judgment and Decision-Making |
| Adam & Shirako (2013) | *JAP* | - Culture - Perceptions of emotional expressiveness - Intention to cooperate or concede in negotiation - Anger expresser’s individual gain | - Ethical, Legal, Diversity, and International Issues - Judgment and Decision-Making - Attitude Theory, Measurement, and Change |
| Adams et al. (2015) | *OBHDP* | - Perceived wrong-doing - Self-righteousness - Avoidance - Resource allocation - Moral self-concepts | - Attitude Theory, Measurement, and Change - Judgment and Decision-Making |
| Antonakis & House (2014) | *LQ* | - Leadership style of supervisor | - Leadership and Management |
| Avery et al. (2015) | *OBHDP* | - Leader race - Leader appraisal - Purchasing intentions | - Ethical, Legal, Diversity, and International Issues - Leadership and Management |
| Barber et al. (2013) | *ORM* | - Random and systematic effects - Insomnia on survey responses | - Research Methods |
| Barber & Budnick (2015) | *JOB* | - Sleepiness - Presence or absence of social threat - Interpretive bias in the workplace | - Attitude Theory, Measurement, and Change |
| Baur (2013) | *Dissertation* | - Demands for OCB displays - Future OCBs and CWBs | - Human Performance |
| Beck & Schmidt (2015) | *JOM* | - Self-efficacy - Time spent on task - Task performance | - Work Motivation - Human Performance |
| Bederensky & Shah (2013) | *AMJ* | - Colleague personality - Task contribution perceptions - Participant task contribution | - Groups and Teams - Attitude Theory, Measurement, and Change - Human Performance |
| Belmi & Neale (2014) | *OBHDP* | - Induced attractiveness or integrity perceptions about self - Perceptions of social class, self-esteem, and power | - Attitude Theory, Measurement, and Change |
| Bhargave et al. (2015) | *OBHDP* | - Job candidate - Number of decision changes - Judgments of hedonic and utilitarian candidate - Choice of hedonic or utilitarian candidate | - Judgment and Decision-Making - Personnel Recruitment, Selection, and Placement |
| Burns et al. (2014) | *JBP* | - Resume cues (biographical information, resume characteristics) - Personality - Perceptions of resume quality | - Attitude Theory, Measurement, and Change - Individual Differences - Personnel Recruitment, Selection, and Placement |
| Burton et al. (2014) | *JOB* | - Perceived reasons (attributions) for abusive supervisor behavior - Aggressive behaviors and citizenship behaviors | - Leadership and Management - Attitude Theory, Measurement, and Change - Occupational Health and Safety - Human Performance |
| Cao et al. (2015) | *ORM* | - Intermediate survey items - Ideal point models | - Statistical Methods/Data Analysis |
| Casciaro et al. (2014) | *ASQ* | - Instrumental social networking for professional goals - Implicit measure of feelings of dirtiness | - Career development - Attitude Theory, Measurement, and Change |
| Cavanaugh et al. (2015) | *OBHDP* | - Personal closeness and gifting role - Appreciation for socially responsible gifts | - Other |
| Caza et al. (2015) | *LQ* | - Follower’s perception of leader - Trust and satisfaction in leader | - Leadership and Management |
| Cheng et al. (2015) | *LQ* | - Country (U.S. vs. Taiwan) - Expectations of supervisor integrity | - Ethical, Legal, Diversity, and International Issues - Leadership and Management |
| Cho & Allen (2012) | *JVB* | - Work interference with family (time and strain) - Trait guilt - Parent-child interactive behavior | - Career Development - Individual Differences |
| Chua (2013) | *AMJ* | - Ambient cultural disharmony - Connecting ideas from disparate cultures - Cultural essentialism - Beliefs about cultural incompatibility - Creativity | - Ethical, Legal, Diversity, and International Issues - Human Performance |
| Clark et al. (2014) | *JBP* | - Work-family coping strategies | - Career Development - Occupational Health and Safety |
| Credé & Harms (2015) | *JOB* | - Confirmatory factor analysis demonstration | - Statistical Methods/Data Analysis |
| Cryder et al. (2013) | *OBHDP* | - Low vs. high perceived impact - Charitable donations | - Other |
| DeKay et al. (2014) | *OBHDP* | - Distortion of information - Pre-decisional distortion - Choice behavior | - Judgment and Decision-Making |
| Desai & Kouchaki (2015) | *OBHDP* | - Reports of units of work completed vs. cost of the same work - Overbilling/unethical behavior | - Ethical, Legal, Diversity, and International Issues - Judgment and Decision-Making |
| Dragoni et al. (2014) | *JAP* | - Exceptional supervisory behaviors | - Leadership and Management |
| Duguid & Thomas-Hunt (2015) | *JAP* | - Prevalence of stereotyping message vs. low prevalence of stereotyping message - Stereotypical perceptions of women | - Ethical, Legal, Diversity, and International Issues - Attitude Theory, Measurement, and Change |
| Dutta & Rao (2015) | *OBHDP* | - Disease contamination anxiety - Cultural contamination concerns | - Ethical, Legal, Diversity, and International Issues |
| DuVernet et al. (2014) | *ORM* | - General mental ability - Response process associated with personality measurement | - Individual Differences |
| Edelman & Larkin (2015) | *OS* | - Position in organizational hierarchy - Status-enhancing deception | - Career Development - Ethical, Legal, Diversity, and International Issues |
| Effron et al. (2015) | *OBHDP* | - Practicing what organization preaches or not - Moral condemnation; perceptions of hypocrisy | - Attitude Theory, Measurement, and Change |
| Effron & Miller (2015) | *OBHDP* | - Suffering vs. non suffering for misdeeds - Perceptions of hypocrisy, self-righteousness - Anger and derogation of advisees - Comfort offering advice | - Attitude Theory, Measurement, and Change |
| Erdogan et al. (2015) | *PPsych* | - Tendency to gossip | - Other |
| Eriksson et al. (2015) | *OBHDP* | - Common-moral association - Dynamics of social norms | - Judgment and Decision-Making |
| Fast et al. (2012) | *OBHDP* | - Experience of power - Overconfident decision-making | - Judgment and Decision-Making |
| Fernbach et al. (2014) | *OBHDP* | - Self control - Performance on a task | - Individual Differences - Human Performance |
| Fine & Pirak (2016) | *JBP* | - Testing conditions - Reaction times - Faking | - Individual Differences |
| Ganegoda & Folder (2015) | *OBHDP* | - Level of counterfactual thinking - Perception of a decision as fair | - Attitude Theory, Measurement, and Change |
| Gladstone & O’Connor (2014) | *OBHDP* | - Facial femininity of counterpart - Negotiator behavior; preference | - Career Development |
| Gu et al. (2014) | *JOB* | - Race, aptitude, and work experience of police officer - Perceived unfairness of an affirmative action decision | - Attitude Theory, Measurement, and Change |
| Guillén et al. (2015) | *LQ* | - Self-to-prototype comparisons in affiliation - Motivation to lead | - Leadership and Management |
| Hardy & Ford (2014) | *ORM* | - Item miscomprehension/ interpretation of scale items | - Research Methods |
| Howell et al. (2015) | *JAP* | - Voice expression - Ethnicity, gender, status - Supervisor voice recognition - Performance evaluation | - Ethical, Legal, Diversity, and International Issues - Leadership and Management - Performance Appraisal/ Management |
| Huang et al. (2015a) | *JBP* | - Survey conditions - Insufficient-effort responding | - Research Methods |
| Inesi & Cable (2015) | *PPsych* | - Competence signals - Gender of subordinates - Performance evaluation | - Performance Appraisal/ Management |
| Johnson et al. (2014) | *JAP* | - Depletion | - Human Performance |
| Juanchich et al. (2012) | *OBHDP* | - Risk quantifiers - Interpretation of risk perception and risk quantifiers | - Judgment and Decision-Making |
| Jung & Lee (2015) | *OBHDP* | - Conflictual vs. harmonious relationship situations in groups - Cognitive persistence - Creativity | - Human Performance - Groups and Teams |
| Kapoutsis et al. (2015) | *JOM* | - Political will | - Individual Differences |
| Karim & Behrend (2014) | *JBP* | - Learner control (instructional, scheduling) - Training reactions - Learning | - Training, Theory, Delivery, Program Design, and Evaluation |
| Karim et al. (2014) | *JBP* | - Testing conditions - Test perceptions - Test performance | - Research Methods - Attitude Theory, Measurement, and Change |
| Kausel et al. (2015) | *OBHDP* | - Narcissim - Advice taking - Perceived competence of person giving advice | - Attitude Theory, Measurement, and Change - Individual Differences |
| Kennedy et al. (2013) | *OBHDP* | - Descriptions of overconfident individuals - Perceptions of overconfidence - Status given | - Attitude Theory, Measurement, and Change |
| Kim et al. (2015) | *OBHDP* | - Social networking - Risk judgment | - Judgment and Decision-Making |
| Koopman et al. (2015) | *OBHDP* | - Employee integration behavior - Leader-member exchange - Justice | - Leadership and Management - Attitude Theory, Measurement, and Change |
| Kouchaki & Desai (2015) | *JAP* | - Anxiety - Self-interested unethical behavior | - Individual Differences - Ethical, Legal, Diversity, and International Issues |
| Kovács et al. (2014) | *OS* | - Perceived authenticity of organization - Consumer value ratings | - Attitude Theory, Measurement, and Change |
| Kray et al. (2014) | *OBHDP* | - Gender differences in the perceived ease of being misled - Likelihood of being deceived in distributive negotiations | - Career Development |
| Lanaj et al. (2014) | *OBHDP* | - Technology use - Sleep quantity/quality - Work engagement - Job control | - Occupational Health and Safety |
| Lazenby & Ansari (2016) | *Conference paper* | - Assertiveness - Leader-member exchange - Gender - Leader outcomes | - Leadership and Management |
| Lee & Gino (2015) | *OBHDP* | - Efforts to regulate aversive affective responses - Decisions in moral dilemmas | - Judgment and Decision-Making |
| Lee et al. (2014) | *JAP* | - Weather - Individual productivity | - Human Performance |
| Lee et al (2015) | *OBHDP* | - Perceived candidate attractiveness - Perceived candidate competence - Selection decisions | - Attitude Theory, Measurement, and Change - Personnel Recruitment, Selection, and Placement |
| Levine & Schweitzer (2015a) | *OBHDP* | - Deception/prosocial lying - Trust | - Attitude Theory, Measurement, and Change |
| Levine & Schweitzer (2015b) | *OBHDP* | - Weight/obesity - Perceptions of competence and warmth - Perceptions of behavioral intentions - Job candidate ratings | - Attitude Theory, Measurement, and Change - Personnel Recruitment, Selection, and Placement |
| Li & Chapman (2013) | *OBHDP* | - Framing effects - Preference for large percentage of a small subset vs. a small percentage of a large subset | - Judgment and Decision-Making |
| Lin-Healy & Small (2012) | *OBHDP* | - Motives for donating to charity - Personality - Prosocial actors - Perceived degree of selfishness | - Attitude Theory, Measurement, and Change |
| Liu et al. (2013) | *OBHDP* | - Other as member of stigmatized social group vs. a non-stigmatized social group - Matching strategy - Desire to avoid hurting members of stigmatized groups | - Attitude Theory, Measurement, and Change - Ethical, Legal, Diversity, and International Issues |
| Long & Christian (2015) | *JAP* | - Mindfulness - Injustice - Ruminative thought - Negative emotions | - Attitude Theory, Measurement, and Change - Occupational Health and Safety |
| Lount et al. (2015) | *OS* | - Team composition - Relationship conflict - Support for diverse teams | - Groups and Teams - Ethical, Legal, Diversity, and International Issues |
| Lowery (2016) | *Thesis* | - Health message exposure - Perceived resource availability - Health-related self-efficacy - Health-related anxiety - Negative/Positive rumination | - Occupational Health and Safety |
| Lyons et al. (2016) | *JOM* | - Visible disabilities - Intentions to manage others’ impressions of visible disabilities; acknowledgment strategies - Claiming and downplaying visible disabilities - Perceived competence and warmth of individuals with visible disabilities - Candidate (with visible disabilities) evaluations | - Ethical, Legal, Diversity, and International Issues - Occupational Health and Safety - Personnel Recruitment, Selection, and Placement |
| Marchiondo et al. (2015) | *LQ* | - Leadership identity construction - Perceived leadership - Decision-making | - Leadership and Management - Judgment and Decision-Making |
| McGonagle et al. (2015) | *JAP* | - Perceived work ability - Antecedents of perceived work ability - Absence, disability, leave, retirement | - Career Development |
| McGonagle & Hamblin (2014) | *JBP* | - Discrimination (perceived, anticipated) - Concealing behaviors - Compensatory behaviors - Job tension | - Ethical, Legal, Diversity, and International Issues |
| Michel et al. (2016) | *JBP* | - Abusive supervision - Trait anger - Aggressive organizational norms - Work related negative affect - Deviance | - Leadership and Management - Individual Differences - Occupational Health and Safety |
| Milkman (2012) | *OBHDP* | - Choice behavior | - Judgment and Decision-Making |
| Milkman et al. (2015) | *JAP* | - Income - Bias toward women and minorities | - Job Evaluation and Compensation |
| Mochon & Frederick (2013) | *OBHDP* | - Boundary conditions of anchoring effects | - Judgment and Decision-Making |
| Moore et al. (2015) | *OBHDP* | - Anchoring - Conversational norms - Naïve intuitive statistics - Over precision in judgment | - Judgment and Decision-Making |
| Mulder & Aquino (2013) | *OBHDP* | - Lying - Threat to self-view of being an honest and moral person | - Other |
| Mulder et al. (2015) | *OBHDP* | - Specific and general rules - Ethical decisions | - Judgment and Decision-Making |
| Nichols & Cottrell (2014) | *LQ* | - Trait desirability in leadership | - Individual Differences - Leadership and Management |
| O’Reilly et al. (2015) | *OS* | - Perceptions of ostracism and harassment | - Attitude Theory, Measurement, and Change |
| Palmeira et al. (2015) | *OBHDP* | - Positive vs. negative outcomes - Attributions of advisor - Attributions of responsibility for the outcome (self vs. advisor) | - Judgment and Decision-Making |
| Parke et al. (2015) | *JAP* | - O*Net coded information processing and creative processing | - Research Methods |
| Parker et al. (2015) | *JBP* | - Trait resilience - Control - Demands - Adaptive coping strategies - Task performance | - Human Performance - Individual Differences |
| Pham et al. (2015) | *OBHDP* | - Affective vs. cognitive system of judgment - Perceived value | - Judgment and Decision-Making |
| Phillips et al. (2014) | *PPsych* | - Recruitment message - Individual differences - Person-job fit - Personal success - Organizational effectiveness | - Career Development - Individual Differences - Personnel Recruitment, Selection, and Placement |
| Pitesa & Thau (2013) | *JAP* | - Perceptions of power | - Attitude Theory, Measurement, and Change |
| Pitesa et al. (2013) | *OBHDP* | - Cognitive control - Interpersonal impact salience - Socially desirable behaviors | - Human Performance |
| Polman (2012) | *OBHDP* | - Choosing for others vs. self - Loss aversion | - Judgment and Decision-Making |
| Polman & Russo (2012) | *OBHDP* | - Internal vs. external attributions - Believing source summoned effort - Predecisional distortion | - Judgment and Decision-Making |
| Porter et al. (2016) | *Journal of Personality* | - Personality - Decisions to initiate working relationships | - Individual Differences |
| Proudfoot et al. (2015) | *OBHDP* | - Economic instability - Employees’ tendency to defensively ignore and diminish organizational problems | - Judgment and Decision-Making - Attitude Theory, Measurement, and Change |
| Quinn & Bunderson (2016) | *JOM* | - Perceived emotions of others | - Attitude Theory, Measurement, and Change |
| Radzevick & Moore (2013) | *OBHDP* | - Comparative judgments - Reaction time - Speed/Efficiency of comparisons | - Judgment and Decision-Making |
| Rai & Diermeier (2015) | *OBHDP* | - Anthropomorphization - Managerial experience - Perceptions of companies - Differences in sympathy | - Attitude Theory, Measurement, and Change |
| Reynolds et al. (2014) | *OBHDP* | - Knowledge of the immorality of an act (moral knowledge) - Moral disengagement | - Other |
| Ritov & Zamir (2014) | *OBHDP* | - Identifiability of those who stand to lose from implementation of affirmative action procedure - Support for hypothetical affirmative action procedures | - Ethical, Legal, Diversity, and International Issues |
| Rosette et al. (2015) | *LQ* | - Perceptions of company names - Leader weakness measure validation | - Research Methods - Attitude Theory, Measurement, and Change - Leadership and Management |
| Rule & Tskhay (2014) | *LQ* | - Faces of American CEOs - Inferences of leadership ability - Company financial performance before and after the 2008 crisis | - Leadership and Management |
| Saqib & Chan (2015) | *OBHDP* | - Time pressure - Risk preferences | - Judgment and Decision-Making |
| Savani & King (2015) | *OBHDP* | - Action construal vs. choice construal vs. event construal - Outcome bias | - Judgment and Decision-Making |
| Savani et al. (2015) | *OBHDP* | - Culture - Normative influence - Adherence to social norms | - Ethical, Legal, Diversity, and International Issues - Judgment and Decision-Making |
| Shah et al. (2015) | *JOM* | - Venting assistance - Problem solving assistance - Learning | - Training Theory, Delivery, Program Design, and Evaluation |
| Sharma et al. (2014) | *OBHDP* | - Financial deprivation - Individual’s moral standards; moral decisions | - Ethical, Legal, Diversity, and International Issues - Judgment and Decision-Making |
| Sherf & Venkataramani (2015) | *OBHDP* | - Positive vs. negative relation to comparison other - Fairness judgments; equity judgments | - Attitude Theory, Measurement, and Change |
| Shirako et al. (2015) | *OBHDP* | - Sympathy; sympathy appeals - Negotiation outcomes | - Judgment and Decision-Making - Attitude Theory, Measurement, and Change |
| Skarlicki & Turner (2014) | *OBHDP* | - Descriptions of job candidates - Justice cues - Gender - Applicant desirability | - Attitude Theory, Measurement, and Change - Personnel Recruitment, Selection, and Placement |
| Spisak et al (2014) | *LQ* | - Age of leaders - Endorsements of stability and change | - Leadership and Management |
| Steffens et al. (2014a) | *LQ* | - Identity Leadership Inventory scale validation | - Leadership and Management - Research Methods |
| Steffens et al. (2014b) | *LQ* | - Shared group membership - Relational identification with leader - Perceived leader charisma - Experience of personal bonding with leader | - Leadership and Management - Attitude Theory, Measurement, and Change |
| Stoughton et al. (2015) | *JBP* | - Employers gaining information about applicants on Facebook - Reactions of job seekers | - Ethical, Legal, Diversity, and International Issues - Attitude Theory, Measurement, and Change |
| Sweldens et al. (2014) | *OBHDP* | - Impression management - Socially undesirable events - Comparative optimism | - Judgment and Decision-Making - Attitude Theory, Measurement, and Change |
| ten Brinke & Adams (2015) | *OBHDP* | - Apologizer’s facial emotion expression - Perception of apologizer’s organization (company confidence; stock market performance) | - Attitude Theory, Measurement, and Change |
| Thau et al. (2015) | *JAP* | - Work group exclusion | - Groups and Teams |
| Tinsley et al. (2015) | *OBHDP* | - Gender - Preference to maximize own wages - Child care expectations - Traditional role orientation - Preference for spouse to earn more - Gender determinism | - Career Development - Attitude Theory, Measurement, and Change |
| Torelli et al. (2014) | *OBHDP* | - Cultural orientation - Status attainment | - Ethical, Legal, Diversity, and International Issues |
| Tost et al. (2015) | *OBHDP* | - Experience of power - Intergenerational decision-making | - Judgment and Decision-Making |
| Uhlmann et al. (2013) | *JOB* | - Nationality - Job references - Evaluation of job candidate | - Ethical, Legal, Diversity, and International Issues - Personnel Recruitment, Selection, and Placement |
| van Dijke et al. (2015) | *OBHDP* | - Nostalgia - Procedural justice - Cooperation | - Attitude Theory, Measurement, and Change |
| van Houwelingen et al. (2017) | *JOM* | - Procedural justice experience - Perceived autonomy - Perceived similarity - Psychological closeness - Procedural fairness enactment (voice) | - Attitude Theory, Measurement, and Change |
| van Houwelingen et al. (2015) | *LQ* | - Leader level of construal mindset - Willingness to enforce moral norms through discipline | - Leadership and Management |
| Walter et al. (2015) | *OS* | - Time spent interacting in the past - Level of engagement - Preferences for reconnecting ties | - Attitude Theory, Measurement, and Change |
| Wan et al. (2013) | *OBHDP* | - Blatant and subtle exposure to idealized female images - Females’ self-evaluations - Attitude towards brands endorsed by the models with these idealized body images | - Attitude Theory, Measurement, and Change |
| Waytz et al. (2015) | *OBHDP* | - Experience of power - Experience of loneliness | - Attitude Theory, Measurement, and Change |
| Welsh & Ordóńez (2014) | *AMJ* | - Subconscious ethical and unethical priming - Subconscious processes on ethical behavior through automatic process of schema activation and implicit association | - Ethical, Legal, Diversity, and International Issues - Judgment and Decision-Making |
| Welsh et al. (2015) | *JAP* | - Moral disengagement - Prevention focus - Unethical behavior | - Ethical, Legal, Diversity, and International Issues |
| Whitson et al. (2015a) | *OBHDP* | - High vs. low job mobility contexts - Social inclusion/exclusion | - Career Development |
| Whitson et al. (2015b) | *OBHDP* | - Recipients vs. observers - Punishment and reward behaviors in organizations | - Judgment and Decision-Making |
| Wiltermuth et al. (2013) | *OBHDP* | - Perceived ethical preferences of others - Own ethical disposition - Making decisions that effect others | - Ethical, Legal, Diversity, and International Issues - Judgment and Decision-Making |
| Wiltermuth & Flynn (2013) | *AMJ* | - Power - Severity of punishment towards transgressors | - Ethical, Legal, Diversity, and International Issues |
| Yam et al. (2014) | *OBHDP* | - Low vs. high social consensus - Ego depletion - Unethical behavior | - Ethical, Legal, Diversity, and International Issues |
| Yam et al. (2014) | *JAP* | - Employee start time - Supervisor chronotype - Supervisor ratings of job performance | - Individual Differences - Performance Appraisal/ Management |
| Young (2016) | *Dissertation* | - Social class - Hiring process outcomes (acquiring, screening, interviewing) | - Personnel Recruitment, Selection, and Placement |
| Yuan (2014) | *Thesis* | - Workplace safety scale development | - Occupational Health and Safety - Research Methods |

**References**

Adam, H., Obodaru, O., and Galinsky, A. D. (2015). Who you are is where you are: Antecedents and consequences of locating the self in the brain or the heart. *Organizational Behavior and Human Decision Processes*. *128*, 74-83. doi:10.1016/j.obhdp.2015.03.004

Adam, H., and Shirako, A. (2013). Not all anger is created equal: The impact of the expresser’s culture on the social effect of anger in negotiations. *Journal of Applied Psychology*. *98*:5, 785-798. doi:10.1037/a0032387

Adams, G. S., Zou, X., Inesi, M. E., and Pillutla, M. M. (2015). Forgiveness is not always divine: When expressing forgiveness makes others avoid you. *Organizational Behavior and Human Decision Processes*. *126*, 130-141. doi:10.1016/j.obhdp.2014.10.003

Antonakis, J., and House, R. J. (2014). Instrumental leadership: Measurement and extension of transformational-transactional leadership theory. *Leadership Quarterly*. *25*, 746-771. doi:10.1016/j.leaqua.2014.04.005

Avery, D. R., McKay, P. F., Volpone, S. D., and Malka, A. (2015). Are companies beholden to bias? The impact of leader race on consumer purchasing behavior. *Organizational Behavior and Human Decision Processes*. *127*, 85-102. doi:10.1016/j.obhdp.2015.01.004

Barber, L. K., Barnes, C. M., and Carlson, K. D. (2013). Random and systematic error effects of insomnia on survey behavior. *Organizational Research Methods*. *16*:4, 616-649. doi:10.1177/1094428113493120

Barber, L. K., and Budnick, C. J. (2015). Turning molehills into mountains: Sleepiness increases workplace interpretive bias. *Journal of Organizational Behavior*. *36*, 360-381. doi:10.1002/job.1992

Baur, J. A. (2013). An investigation of OCB demands and workplace behaviors. [dissertation]. [Tampa (FL)]: University of South Florida

Beck, J. W., and Schmidt, A. M. (2015). Negative relationships between self-efficacy and performance can be adaptive: The mediating role of resource allocation. *Journal of Management*. Advance online publication. doi:10.1177/0149206314567778

Bederensky, C., and Shah, N. P. (2013). The downfall of extraverts and rise of neurotics: The dynamic process of status allocation in task groups. *Academy of Management Journal*. *56*:2, 387-406. doi:10.5465/amj.2011.0316

Belmi, P., and Neale, M. (2014). Mirror, mirror on the wall, who’s the fairest of them all? Thinking that one is attractive increases the tendency to support inequality. *Organizational Behavior and Human Decision Processes*. *124*, 133-149. doi:10.1016/j.obhdp.2014.03.002

Bhargave, R., Chakravarti, A., and Guha, A. (2015). Two-stage decisions increase preference for hedonic options. *Organizational Behavior and Human Decision Processes*. *130*, 123-135. doi:10.1016/j.obhdp.2015.06.003

Burns, G. N., Christiansen, N. D., Morris, M. B., Periard, D. A., and Coaster, J. A. (2014). Effects of applicant personality on resume evaluations. *Journal of Business and Psychology*. *29*, 573-591. doi:10.1007/s10869-014-9349-6

Burton, J. P., Taylor, S. G., and Barber, L. K. (2014). Understanding internal, external, and relational attributions for abusive supervision. *Journal of Organizational Behavior*. *35*, 871-891. doi:10.1002/job.1939

Cao, M., Drasgow, F., and Cho, S. (2015). Developing ideal intermediate personality items for the ideal point model. *Organizational Research Methods*. *18*:2, 252-275. doi:10.1177/1094428114555993

Casciaro, T., Gino, F., and Kouchaki, M. (2014). The contaminating effects of building instrumental ties: How networking can make us feel dirty. *Administrative Science Quarterly*. *59*:4, 705-735. doi:10.1177/0001839214554990

Cavanaugh, L. A., Gino, F., and Fitzsimons, G. J. (2015). When doing good is bad in gift giving: Mis-predicting appreciation of socially responsible gifts. *Organizational Behavior and Human Decision Processes*. *131*, 178-189. doi:10.1016/j.obhdp.2015.07.002

Caza, A., Zhang, G., Wang, L., and Bai, Y. (2015). How do you really feel? Effect of leaders’ perceived emotional sincerity on followers’ trust. *Leadership Quarterly*. *26*, 518-531. doi:10.1016/j.leaqua.2015.05.008

Cheng, C. –Y., Jiang, D. –Y., Cheng, B. –S., Riley, J. H., and Jen, C. –K. (2015). When do subordinates commit to their supervisors? Different effects of perceived supervisor integrity and support on Chinese and American employees. *Leadership Quarterly*. *26*, 81-97. doi:10.1016/j.leaqua.2014.08.002

Cho, E., and Allen, T. D. (2012). Relationship between work interference with family and parent-child interactive behavior: Can guilt help? *Journal of Vocational Behavior*. *80*, 276-287. doi:10.1016/j.jvb.2011.12.002

Chua, R. Y. J. (2013). The costs of ambient cultural disharmony: Indirect intercultural conflicts in social environment undermine creativity. *Academy of Management Journal*. *56*:6, 1545-1577. doi:10.5465/amj.2011.0971

Clark, M. A., Michel, J. S., Early, R. J., and Baltes, B. B. (2014). Strategies for coping with work stressors and family stressors: Scale development and validation. *Journal of Business and Psychology*. *29*, 617-638. doi:10.1007/s10869-014-935607

Credé, M., and Harms, P. D. (2015). 25 years of higher-order confirmatory factor analysis in the organizational sciences: A critical review and development of reporting recommendations. *Journal of Organizational Behavior*. *36*, 845-872. doi:10.1002/job.2008

Cryder, C. E., Loewenstein, G., and Scheines, R. (2013). The donor is in the details. *Organizational Behavior and Human Decision Processes*. *120*, 15-23. doi:10.1016/j.obhdp.2012.08.002

DeKay, M. L., Miller, S. A., Schley, D. R., and Erford, B. M. (2014). Proleader antitrailer information distortion and their effects on choice and post choice memory. *Organizational Behavior and Human Decision Processes*. *125*, 134-150. doi:10.1016/j.obhdp.2014.07.003

Desai, S. D., and Kouchaki, M. (2015). Work-report formats and overbilling: How unit-reporting vs. cost-reporting increases accountability and decreases overbilling. *Organizational Behavior and Human Decision Processes*. *130*, 79-88. doi:10.1016/j.obhdp.2015.06.007

Dragoni, L., Park, H., Soltis, J., and Forte-Trammell, S. (2014). Show and tell: How supervisors facilitate leader development among transitioning leaders. *Journal of Applied Psychology*. *99*:1, 66-86. doi:10.1037/a0034452

Duguid, M. M., and Thomas-Hunt, M. C. (2015). Condoning stereotyping? How awareness of stereotyping prevalence impacts expression of stereotypes. *Journal of Applied Psychology*. *100*:2, 343-359. doi:10.1037/a0037908

Dutta, S., and Rao, H. (2015). Infectious diseases, contamination rumors, and ethnic violence: Regimental mutinies in the Bengal Native Army in 1857 India. *Organizational Behavior and Human Decision Processes*. *127*, 36-47. doi:10.1016/j.obhdp.2014.10.004

DuVernet, A. M., Wright, N. A., Meade, A. W., Coughlin, C., and Kantrowitz, T. M. (2014). General mental ability as a source of differential functioning in personality scales. *Organizational Research Methods.* *17*:3, 299-323. doi:10.1177/109442811425996

Edelman, B., and Larkin, I. (2015). Social comparisons and deception across workplace hierarchies: Field and experimental evidence. *Organization Science*. *26*:1, 78-98. doi:10.1287/orsc.2014.0938

Effron, D. A., Lucas, B. J., and O’Connor, K. (2015). Hypocrisy by association: When organizational membership increases condemnation for wrongdoing. *Organizational Behavior and Human Decision Processes*. *130*, 147-159. doi:10.1016/j.obhdp.201505.001

Effron, D. A., and Miller, D. T. (2015). Do as I say, not as I’ve done: Suffering for a misdeed reduces the hypocrisy of advising others against it. *Organizational Behavior and Human Decision Processes*. *131*, 16-32. doi:10.1016/j.obhdp.2015.07.004

Erdogan, B., Bauer, T. N., and Walter, J. (2015). Deeds that help and words that hurt: Helping and gossip as moderators of the relationship between leader-member exchange and advice network centrality. *Personnel Psychology*. *68*, 185-214. doi:10.1111/peps.12075

Eriksson, K., Strimling, P., and Coultas, J. C. (2015). Bidirectional associations between descriptive and injunctive norms. *Organizational Behavior and Human Decision Processes*. *127*, 59-69. doi:10.1016/j.obhdp.2014.09.011

Fast, N. J., Sivanathan, N., Mayer, N. D., and Galinsky, A. D. (2012). Power and overconfident decision-making. *Organizational Behavior and Human Decision Processes*. *117*, 249-260. doi:10.1016/j.obhdp.2011.11.009

Fine, S., and Pirak, M. (2016). Faking fast and slow: Within-person response time latencies for measuring faking in personnel testing. *Journal of Business and Psychology*. *31*, 51-64. doi:10.1007/s10869-0159398-5

Ganegoda, D. B., and Folder, R. (2015). Framing effects in justice perceptions: Prospect theory and counterfactuals. *Organizational Behavior and Human Decision Processes*. *126*, 27-36. doi:10.1016/j.obhdp.2014.10.002

Gladstone, E., and O’Connor, K. M. (2014). A counterpart’s famine face signals cooperativeness and encourages negotiators to compete. *Organizational Behavior and Human Decision Processes*. *125*, 18-25. doi:10.1016/j.obhdp.2014.05.001

Gu, J., McFerran, B., Aquino, K., and Kim, T. G. (2014). What makes affirmative action-based hiring decisions seem (un)fair?: A test of an ideological explanation for fairness judgments. *Journal of Organizational Behavior*. *35*, 722-745. doi:10.1002/job.1927

Guillén, L., Mayo, M., and Korotov, K. (2015). Is leadership a part of me? A leader identify approach to understanding the motivation to lead. *Leadership Quarterly*. *26*, 802-820. doi:10.1016/j.leaqua.2015.05.001

Hardy, B., and Ford, L. R. (2014). It’s not me, it’s you: Miscomprehension in surveys. *Organizational Research Methods*. *17*:2, 138-162. doi:10.1177/1094428113520185

Howell, T. M., Harrison, D. A., Burris, E. R., and Detert, J. R. (2015). Who gets credit for input? Demographic and structural status cues in voice recognition. *Journal of Applied Psychology*. *100*:6, 1765-1784. doi:10.1037/ap10000025

Huang, J. L., Bowling, N. A., Liu, M., and Li, Y. (2015a). Detecting insufficient effort responding with an infrequency scale: Evaluating validity and participant reaction. *Journal of Business and Psychology*. *30*, 299-311. doi:10.1007/s10869-014-9357-6

Inesi, M., E., and Cable, D. M. (2015). When accomplishments come back to haunt you: The negative effect of competence signals on women’s performance evaluations. *Personnel Psychology*. *68*, 615-657. doi:10.1111/peps.12083

Johnson, R. E., Lanaj, K., and Barnes, C. M. (2014). The good and bad of being fair: Effects of procedural and interpersonal justice behaviors on regulatory resources. *Journal of Applied Psychology*. *99*:4, 635-650. doi:10.1037/a0035647

Juanchich, M., Sirota, M., and Butler, C. L. (2012). The perceived functions of linguistic risk quantifiers and their effect on risk, negativity perception and decision making. *Organizational Behavior and Human Decision Processes*. *118*, 72-81. doi:10.1016/j.obhdp.2012.01.002

Jung, E. J., and Lee, S. (2015). The combined effects of relationship conflict and the relational self on creativity. *Organizational Behavior and Human Decision Processes*. *130*, 44-57. doi:10.1016/j.obhdp.2015.06.006

Kapoutsis, I., Papalexandris, A., Treadway, D. C., and Bentley, J. (2015). Measuring political will in organizations: Theoretical construct development and empirical validation. *Journal of Management*. Advance online publication. doi:10.1177/0149206314566460

Karim, M. N., and Behrend, T. S. (2014). Reexamining the nature of learner control: Dimensionality and effects of learning and training reactions. *Journal of Business and Psychology*. *29*, 87-99. doi:10.1007/s10869-013-9309-6

Karim, M. N., Kaminsky, S. E., and Behrend, T. S. (2014). Cheating, reactions, and performance in remotely proctored testing: An exploratory experimental study. *Journal of Business and Psychology*. *29*, 555-572. doi:10.1007/s10869-014-9343-z

Kausel, E. E., Culbertson, S. S., Leiva, P. I., Slaughter, J. E., and Jackson, A. T. (2015). Too arrogant for their own good? Why and when narcissists dismiss advice. *Organizational Behavior and Human Decision Processes*. *131*, 33-50. doi:10.1016/j.obhdp.2015.07.006

Kennedy, J. A., Anderson, C., and Moore, D. A. (2013). When overconfidence is revealed to others: Testing the status-enhancement theory of overconfidence. *Organizational Behavior and Human Decision Processes*. *122*, 266-279. doi:10.1016/j.obhdp.2013.08.005

Kim, H., Lee, K., and Park, K. (2015). Balancing out feelings of risk by playing it safe: The effect of social networking on subsequent risk judgment. *Organizational Behavior and Human Decision Processes*. *131*, 121-131. doi:10.1016/j.obhdp.2015.09.002

Koopman, J., Matta, F. K., Scott, B. A., and Conlon, D. E. (2015). Ingratiation and popularity as antecedents of justice: A social exchange and social capital perspective. *Organizational Behavior and Human Decision Processes*. *131*, 132-148. doi:10.1016/j.obhdp.2015.09.001

Kouchaki, M., and Desai, S. D. (2015). Anxious, threatened, and also unethical: How anxiety makes individuals feel threatened and commit unethical acts. *Journal of Applied Psychology*. *100*:2, 360-375. doi:10.1037/a0037796

Kovács, B., Carroll, G. R., and Lehman, D. W. (2014). Authenticity and consumer value ratings: Empirical tests from the restaurant domain. *Organization Science*. *25*:2, 458-478. doi:10.1287/orsc.2013.0843

Kray, L. J., Kennedy, J. A., and Van Zant, A. B. (2014). Not competent enough to know the difference? Gender stereotypes about women’s ease of being misled predict negotiator deception. *Organizational Behavior and Human Decision Processes*. *125*, 61-72. doi:10.1016/j.obhdp.2014.06.002

Lanaj, K., Johnson, R. E., and Barnes, C. M. (2014). Beginning the workday yet already depleted? Consequences of late-night smartphone use and sleep. *Organizational Behavior and Human Decision Processes*. *124*, 11-23. doi:10.1016/j.obhdp.2014.01.001

Lazenby, C. S., and Ansari, M. A. (2016, April). *Assertiveness and leadership perceptions: The role of gender and LMX*. Paper presented at the 31^st^ Annual Meeting of the Society for Industrial and Organizational Psychology, Anaheim, CA.

Lee, J. J., and Gino, F. (2015). Poker-faced morality: Concealing emotions leads to utilitarian decision making. *Organizational Behavior and Human Decision Processes*. *126*, 49-64. doi:10.1016/j.obhdp.2014.10.006

Lee, J. J., Gino, F., and Staats, B. R. (2014). Rainmakers: Why bad weather means good productivity. *Journal of Applied Psychology*. *99*:3, 504-513. doi:10.1037/a0035559

Lee, S., Pitesa, M., Pillutla, M., and Thau, S. (2015). When beauty helps and when it hurts: An organizational context model of attractiveness discrimination in selection decisions. *Organizational Behavior and Human Decision Processes*. *128*, 15-28. doi:10.1016/j.obhdp.2015.02.003

Levine, E. E., and Schweitzer, M. E. (2015a). Prosocial lies: When deception breeds trust. *Organizational Behavior and Human Decision Processes*. *126*, 88-106. doi:10.1016/j.obhdp.2014.10.007

Levine, E. E., and Schweitzer, M. E. (2015b). The affective and interpersonal consequences of obesity. *Organizational Behavior and Human Decision Processes*. *127*, 66-84. doi:10.1016/j.obhdp.2015.01.002

Li, M., and Chapman, G. B. (2013). A big fish or a small pond? Framing effects on percentages. *Organizational Behavior and Human Decision Processes*. *122*, 190-199. doi:10.1016/j.obhdp.2013.07.003

Lin-Healy, F., and Small, D. A. (2012). Cheapened altruism: Discounting personally affected prosocial actors. *Organizational Behavior and Human Decision Processes*. *117*, 269-274. doi: 10.1016/j.obhdp.2011.11.006

Liu, P. J., Campbell, T. H., Fitzsimons, G. J., and Fitzsimons, G. M. (2013). Matching choices to avoid offending stigmatized group members. *Organizational Behavior and Human Decision Processes*. *122*, 291-304. doi:10.1016/j.obhdp.2013.08.007

Long, E. C., and Christian, M. S. (2015). Mindfulness buffers retaliatory responses to injustice: A regulatory approach. *Journal of Applied Psychology*. *100*:5, 1409-1422. doi:10.1037/apl0000019

Lount, R. B., Sheldon, O. J., Rink, F., and Phillips, K. W. (2015). Biased perceptions of racially diverse teams and their consequences for resource support. *Organization Science*. *26*:5, 1351-1364. doi:10.1287/orsc.2015.0994

Lowery, M. R. (2016). The role of resources and message exposure in health-related outcomes. [master’s thesis]. [Greenville (NC)]: East Carolina University.

Lyons, B. J., Martinez, L. R., Ruggs, E. N., Hebl, M. R., Ryan, A. M., O’Brien, K. R., and Roebuck, A. (2016). To say or not to say: Different strategies for acknowledging a visible disability. *Journal of Management*. Advance online publication. doi:10.1177/0149206316638160

Marchiondo, L. A., Myers, C. G., and Kopelman, S. (2015). The relational nature of leadership identity construction: How and when it influences perceived leadership and decision-making. *Leadership Quarterly*. *26*, 892-908. doi:10.1016/j.leaqua.2015.06.006

McGonagle, A. K., Fisher, G. G., Barnes-Farrell, J. L., and Grosch, J. W. (2015). Individual and work factors related to perceived work ability and labor force outcomes. *Journal of Applied Psychology*. *100*:2, 376-398. doi:10.1037/a0037974

McGonagle, A. K., and Hamblin, L. E. (2014). Proactive responding to anticipated discrimination based on chronic illness: Double-edged sword? *Journal of Business and Psychology*. *29*, 427-442. doi:10.1007/s10869-013-9324-7

Michel, J. S., Newness, K., and Duniewicz, K. (2016). How abusive supervision affects workplace deviance: A moderated-mediation examination of aggressiveness and work-related negative affect. *Journal of Business and Psychology*. *31*, 1-22. doi:10.1007/s10869-015-9400-2

Milkman, K. L. (2012). Unsure what the future will bring? You may overindulge: Uncertainty increases the appeal of *wants* over *should*. *Organizational Behavior and Human Decision Processes*. *119*, 163-176. doi:10.1016/j.obhdp.2012.17.003

Milkman, K. L., Akinola, M., and Chugh, D. (2015). What happens before? A field experiment exploring how pay and representation differently shape bias on the pathway into organizations. *Journal of Applied Psychology*. *100*:6, 1678-1712. doi:10.1037/ap10000022

Mochon, D., and Frederick, S. (2013). Anchoring in sequential judgments. *Organizational Behavior and Human Decision Processes*. *122*, 69-79. doi:10.1016/j.obhdp.2013.04.002

Moore, D. A., Carter, A. B., and Yang, H. H. J. (2015). Wide of the mark: Evidence on the underlying causes of overprecision in judgment. *Organizational Behavior and Human Decision Processes*. *131*, 110-120. doi:10.1016/j.obhdp.2015.09.003

Mulder, L. B., and Aquino, K. (2013). The role of moral identity in the aftermath of dishonesty. *Organizational Behavior and Human Decision Processes*. *121*, 219-230. doi:10.1016/j.obhdp.2013.03.005

Mulder, L. B., Jordan, J., and Rink, F. (2015). The effect of specific and general rules on ethical decisions. *Organizational Behavior and Human Decision Processes*. *126*, 115-129. doi:10.1016/j.obhdp.2014.11.002

Nichols, A. L., and Cottrell, C. A. (2014). What do people desire in their leaders? The role of leadership level on trait desirability. *Leadership Quarterly*. *25*, 711-729. doi:10.1016/j.leaqua.2014.04.001

O’Reilly, J., Robinson, S. L., Berdahl, J. L., and Banki, S. (2015). Is negative attention better than no attention? The comparative effects of ostracism and harassment at work. *Organization Science*. *26*:3, 774-793. doi:10.1287/orsc.2014.0900

Palmeira, M., Spassova, G., and Keh, H. T. (2015). Other-serving bias in advice taking: When advisors receive more credit than blame. *Organizational Behavior and Human Decision Processes*. *130*, 13-25. doi:10.1016/j.obhdp.2015.06.001

Parke, M. R., Seo, M. –G., and Sherf, E. N. (2015). Regulating and facilitating: The role of emotional intelligence in maintaining and using positive affect for creativity. *Journal of Applied Psychology*. *100*:3, 917-934. doi:10.1037/a0038452

Parker, S. L., Jimmieson, N. L., Walsh, A. J., and Loakes, J. L. (2015). Trait resilience fosters adaptive coping when control opportunities are high: Implications for the motivating potential of active work. *Journal of Business and Psychology*. *30*:3, 583-604. doi:10.1007/s10869-014-9383-4

Pham, M. T., Faraji-Rad, A., Toubia, O., and Lee, L. (2015). Affect as an ordinal system of utility assessment. *Organizational Behavior and Human Decision Processes*. *131*, 81-94. doi:10.1016/j.obhdp.2015.08.003

Phillips, J. M., Gully, S. M., McCarthy, J. E., Castellano, W. G., and Kim, M. S. (2014). Recruiting global travelers: The role of global travel recruitment messages and individual differences in perceived fit, attraction, and job pursuit intentions. *Personnel Psychology*. *67*, 153-201. doi:10.1111/peps.12043

Pitesa, M., and Thau, S. (2013). Masters of the universe: How power and accountability influence self-serving decisions under moral hazard. *Journal of Applied Psychology*. *98*:3, 550-558. doi:10.1037/a0031697

Pitesa, M., Thau, S., and Pillutla, M. M. (2013). Cognitive control and socially desirable behavior: The role of interpersonal impact. *Organizational Behavior and Human Decision Processes*. *122*, 232-243. doi:10.1016/j.obhdp.2013.08.003

Polman, E. (2012). Self-other decision making and loss aversion. *Organizational Behavior and Human Decision Processes*. *119*, 141-150. doi:10.1016/j.obhdp.2012.06.005

Polman, E., and Russo, J. E. (2012). Commitment to a developing preference and predecisional distortion of information. *Organizational Behavior and Human Decision Processes*. *119*, 78-88. doi:10.1016/j.obhdp.2012.05.004

Porter, C., Parrigon, S., Woo, S. E., Saef, R., and Tay, L. (2016). Cultural and intellectual openness differentially relate to social judgments of potential work partners. *Journal of Personality*. Advance online publication. doi:10.1111/jopy.12266

Proudfoot, D., Kay, A. C., and Mann, H. (2015). Motivated employee blindness: The impact of labor market instability on judgment of organizational inefficiencies. *Organizational Behavior and Human Decision Processes*. *130*, 108-122. doi:10.1016/j.obhdp.2015.06.008

Quinn, R. W., and Bunderson, J. S. (2016). Could we huddle on this project? Participant learning in newsroom conversations. *Journal of Management*. *42*:2, 386-418. doi:10.1177/0149206313484517

Radzevick, J. R., and Moore, D. A. (2013). Just how comparative are comparative judgments. *Organizational Behavior and Human Decision Processes*. *122*, 80-91. doi:10.1016/j.obhdp.2013.05.001

Rai, T. S., and Diermeier, D. (2015). Corporations are Cyborgs: Organizations elicit anger but not sympathy when they can think but cannot feel. *Organizational Behavior and Human Decision Processes*. *126*, 18-26. doi:10.1016/j.obhdp.2014.10.001

Reynolds, S. J., Dang, C. T., Yam, K. C., and Leavitt, K. (2014). The role of moral knowledge in everyday immorality: What does it matter if I know what is right?. *Organizational Behavior and Human Decision Processes*. *123*, 124-137. doi:10.1016/j.obhdp.2013.10.008

Ritov, I., and Zamir, E. (2014). Affirmative action and other group tradeoff policies: Identifiability of those adversely affected. *Organizational Behavior and Human Decision Processes*. *125*, 50-60. doi:10.1016/j.obhdp.2014.04.002

Rosette, A. S., Mueller, J. S., and Lebel, R. D. (2015). Are male leaders penalized for seeking help? The influence of gender and asking behaviors on competence perceptions. *Leadership Quarterly*. *26*, 749-762. doi:10.1016/j.leaqua.2015.02.001

Rule, N. O., and Tskhay, K. O. (2014). The influence of economic context on the relationship between chief executive officer facial appearance and company profits. *Leadership Quarterly*. *25*, 846-854. doi:10.1016/j.leaqua.2014.01.001

Saqib, N. U., and Chan, E. Y. (2015). Time pressure reverses risk preferences. *Organizational Behavior and Human Decision Processes*. *130*, 58-68. doi:10.1016/j.obhdp.2015.06.004

Savani, K., and King, D. (2015). Perceiving outcomes as determined by external forces: The role of event construal in attenuating the outcome bias. *Organizational Behavior and Human Decision Processes*. *130*, 136-146. doi:10.1016/j.obhdp.2015.05.002

Savani, K., Wadhwa, M., Uchida, Y., Ding, Y., and Naidu, N. V. R. (2015). When norms loom larger than the self: Susceptibility of preference-choice consistency to normative influence across cultures. *Organizational Behavior and Human Decision Processes*. *129*, 70-79. doi:10.1016/j.obhdp.2014.09.001

Shah, N. P., Cross, R., and Levin, D. Z. (2015). Performance benefits from providing assistance in networks: Relationships that generate learning. *Journal of Management*. Advance online publication. doi:10.1177/0149206315584822

Sharma, E., Mazar, N., Alter, A. L., and Ariely, D. (2014). Financial deprivation selectivity shifts moral standards and compromises moral decisions. *Organizational Behavior and Human Decision Processes*. *123*, 90-100. doi:10.1016/j.obhdp.2013.09.001

Sherf, E. N., and Venkataramani, V. (2015). Friend or foe? The impact of relational ties with comparison others on outcome fairness and satisfaction judgments. *Organizational Behavior and Human Decision Processes*. *128*, 1-14. doi:10.1016/j.obhdp.2015.02.002

Shirako, A., Kilduff, G. J., and Kray, L. J. (2015). Is there a place for sympathy in negotiation? Finding strength in weakness. *Organizational Behavior and Human Decision Processes*. *131*, 95-109. doi:10.1016/j.obhdp.2015.09.004

Skarlicki, D. P., and Turner, R. A. (2014). Unfairness begets unfairness: Victim derogation bias in employee ratings. *Organizational Behavior and Human Decision Processes*. *124*, 34-46. doi:10.1016/j.obhdp.2013.11.004

Spisak, B. R., Grabo, A. E., Arvey, R. D., and van Vugt, M. (2014). The age of exploration and exploitation: Younger-looking leaders endorsed for change and older-looking leaders endorsed for stability. *Leadership Quarterly*. *25*, 805-816. doi:10.1016/j.leaqua.2014.06.001

Steffens, N. K., Haslam, S. A., Reicher, S. D., Platow, M. J., Fransen, K., Yang, J, … Boen, F. (2014a). Leadership as social identity management: Introducing the Identity Leadership Inventory (ILI) to assess and validate a four-dimensional model. *Leadership Quarterly*. *25*, 1001-1024. doi:10.1016/j.leaqua.2014.05.002

Steffens, N. K., Haslam, S. A., and Reicher, S. D. (2014b). Up close and personal: Evidence that shared social identity is a basis for the ‘special’ relationship that binds followers to leaders. *Leadership Quarterly*. *25*, 296-313. doi:10.1016/j.leaqua.2013.08.008

Stoughton, J. W., Thompson, L. F., and Meade, A. W. (2015). Examining applicant reactions to the use of social networking websites in pre-employment screening. *Journal of Business and Psychology*. *30*, 73-88. doi:10.1007/s10869-013-9333-6

Sweldens, S., Puntoni, S., Paolacci, G., and Vissers, M. (2014). The bias in the bias: Comparative optimism as a function of event social undesirability. *Organizational Behavior and Human Decision Processes*. *124*, 229-244. doi:10.1016/j.obhdp.2014.03.007

ten Brinke, L., and Adams, G. S. (2015). Saving face? When emotion displays during public apologies mitigate damage to organizational performance. *Organizational Behavior and Human Decision Processes*. *130*, 1-12. doi:10.1016/j.obhdp.2015.05.003

Thau, S., Derfler-Rozin, R., Pitesa, M., Mitchell, M. S., and Pillutla, M. M. (2015). Unethical for the sake of the group: Risk of social exclusion and pro-group unethical behavior. *Journal of Applied Psychology*. *100*:1, 98-113. doi:10.1037/a0036708

Tinsley, C. H., Howell, T. M., and Amanatullah, E. T. (2015). Who should bring home the bacon? How deterministic views of gender constrain spousal wage preferences. *Organizational Behavior and Human Decision Processes*. *126*, 37-48. doi:10.1016/j.obhdp.2014.09.003

Torelli, C. J., Leslie, L. M., Stoner, J. L., and Puente, R. (2014). Cultural determinants of status: Implications for workplace evaluations and behaviors. *Organizational Behavior and Human Decision Processes*. *123*, 34-48. doi:10.1016/j.obhdp.2013.11.001

Tost, L. P., Wade-Benzoni, K. A., and Johnson, H. H. (2015). Noblesse oblige emerges (with time): Power enhances intergenerational beneficence. *Organizational Behavior and Human Decision Processes*. *128*, 61-73. doi:10.1016/j.obhdp.2015.03.003

Uhlmann, E. L., Heaphy, E., Ashford, S. J., Zhu, L., and Sanchez-Burks, J. (2013). Acting professional: An exploration of culturally bounded norms against nonwork role referencing. *Journal of Organizational Behavior*. *34*, 866-886. doi:10.1002/job.1874

van Dijke, M., Wildschut, T., Leuissen, J. M., and Sedikides, C. (2015). Nostalgia buffers the negative impact of low procedural justice on cooperation. *Organizational Behavior and Human Decision Processes*. *127*, 15-29. doi:10.1016/j.obhdp.2014.11.005

van Houwelingen, van Dijke, M., and De Cremer, D. (2017). Fairness enactment as response to higher level unfairness: The roles of self-construal and spatial distance. *Journal of Management*. *43*:2, 319-348. doi:10.1177/0149206314530166

van Houwelingen, van Dijke, M., and De Cremer, D. (2015). Getting it done and getting it right: Leader disciplinary reactions to Followers’ moral transgressions are determined by construal level mindset. *Leadership Quarterly*. *26*, 878-891. doi:10.1016/j.leaqua.2015.06.007

Walter, J., Levin, D. Z., and Murnighan, J. K. (2015). Reconnection choices: Selecting the most valuable (vs. most preferred) dormant ties. *Organization Science*. *26*:5, 1447-1465. doi:10.1287/orsc.2015.0996

Wan, F., Ansons, T. L., Chattopadhyay, A., and Leboe, J. P. (2013). Defensive reactions to slim female images in advertising: The moderating role of mode of exposure. *Organizational Behavior and Human Decision Processes*. *120*, 37-46. doi:10.1016/j.obhdp.2012.07.008

Waytz, A., Chou, E. Y., Magee, J. C., and Galinsky, A. D. (2015). Not so lonely at the top: The relationship between power and loneliness. *Organizational Behavior and Human Decision Processes*. *130*, 69-78. doi:10.1016/j.obhdp.2015.06.002

Welsh, D. T., and Ordóñez, L. D. (2014). Conscience without cognition: The effects of subconscious priming on ethical behavior. *Academy of Management Journal*. *57*:3, 723-742. doi:10.5465/amj.2011.1009

Welsh, D. T., Ordóñez, L. D., Snyder, D. G., and Christian, M. S. (2015). The slippery slope: How small ethical transgressions pave the way for larger future transgressions. *Journal of Applied Psychology*. *100*:1, 114-127. doi:10.1037/a0036950

Whitson, J. A., Wang, C. S., Kim, J., Cao, J., and Scrimpshire, A. (2015a). Responses to normative and norm-violating behavior: Culture, job mobility, and social inclusion and exclusion. *Organizational Behavior and Human Decision Processes*. *127*, 24-35. doi:10.1016/j.obhdp.2014.08.001

Whitson, J. A., Wang, C. S., See, Y. H. M., Baker, W. E., and Murnighan, J. K. (2015b). How, when, and why recipients and observers reward good deeds and punish bad deeds. *Organizational Behavior and Human Decision Processes*. *128*, 84-95. doi:10.1016/j.obhdp.2015.03.006

Wiltermuth, S. S., Bennett, V. M., and Pierce, L. (2013). Doing as they would do: How the perceived ethical preferences of third-party beneficiaries impact ethical decision-making. *Organizational Behavior and Human Decision Processes*. *122*, 280-290. doi:10.1016/j.obhdp.2013.10.001

Wiltermuth, S. S., and Flynn, F. J. (2013). Power, moral clarity, and punishment in the workplace. *Academy of Management Journal*. *56*:4, 1002-1023. doi:10.5465/amj.2010.0960

Yam, K. C., Chen, X., -P., and Reynolds, S. J. (2014). Ego depletion and its paradoxical effects on ethical decision making. *Organizational Behavior and Human Decision Processes*. *124*, 204-214. doi:10.1016/j.obhdp.2014.03.008

Yam, K. C., Fehr, R., and Barnes, C. M. (2014). Morning employees are perceived as better employees: Employees’ start times influence supervisor performance ratings. *Journal of Applied Psychology*. *99*:6, 1288-1299. doi:10.1037/a0037109

Young, N. C. J. (2016). The impact of social class in the hiring process. [dissertation]. [Storrs (CT)]: University of Connecticut

Yuan, Z. (2014). A preliminary development and validation of a measure of safety performance. [master’s thesis]. [Indianapolis (IN)]: Purdue University.
